# A Multifunctional Cobalt‐Containing Implant for Treating Biofilm Infections and Promoting Osteointegration in Infected Bone Defects Through Macrophage‐Mediated Immunomodulation

**DOI:** 10.1002/advs.202409200

**Published:** 2024-11-26

**Authors:** Nongyang Yan, Hao Zhou, Penghe Jin, Tengfei Li, Qi Liu, Hao Ning, Zhixin Ma, Linfei Feng, Tao Jin, Youwen Deng, Zhengwei Wu

**Affiliations:** ^1^ Institute of Advanced Technology University of Science and Technology of China No. 5089 Wangjiang West Road Hefei Anhui 230031 China; ^2^ Department of Spine Surgery The Third Xiangya Hospital Central South University No. 138 Tongzi Road Changsha Hunan 410013 China; ^3^ School of Nuclear Science and Technology University of Science and Technology of China No. 96 Jinzhai Road Hefei Anhui 230026 China; ^4^ Comprehensive supervision office Anhui provincial Health Commission 435 Tunbrook Road Hefei 230032 China; ^5^ Department of Oral and Maxillofacial Surgery The First Affiliated Hospital of Anhui Medical University No. 218 Jixi Avenue Heifei Anhui 230032 China

**Keywords:** bone infection therapy, cobalt‐containing implants, immunomodulation, osteogenic differentiation, plasma immersion ion implantation (PIII)

## Abstract

Treating bone infections and ensuring bone recovery is one of the major global problems facing modern orthopedics. Prolonged antibiotic use may increase the risk of antimicrobial resistance, and inflammation caused by biofilms can obstruct tissue healing, making bone infection treatment even more challenging. The optimal treatment strategy combines immune response modification to promote osteogenesis with effective bacterial infection removal that does not require long‐term antibiotic use. A one‐step plasma immersion ion implantation approach is used to create titanium alloy implants incorporating cobalt. According to experimental findings, cobalt‐containing titanium implants exhibit improved antibacterial activity by efficiently disrupting biofilm formations and reducing Methicillin‐resistant *Staphylococcus aureus* adherence by over 80%. Additionally, the implants exhibit superior anti‐inflammatory and osseointegration properties. RNA sequencing analysis reveals the potential mechanism of Co^2+^ in regulating the polarization of macrophages toward the anti‐inflammatory M2 phenotype, which is crucial for creating an immune environment conducive to bone healing. Concurrently, these implants promote osteogenic differentiation while suppressing osteoclast activity, further supporting bone repair. Overall, without exogenous recombinant proteins or antibiotics, the implants effectively eradicate infections and expedite bone repair, offering a novel therapeutic strategy for complex skeletal diseases with clinical promise.

## Graphical abstract

This study introduces cobalt‐titanium implants via a one‐step plasma immersion ion implantation method for bone infection treatment. These implants enhance antibacterial action, reduce inflammation, improve osseointegration, and facilitate bone healing without antibiotics or recombinant proteins. RNA sequencing elucidates Co^2+^'s role in macrophage polarization towards the M2 phenotype, presenting a potential therapeutic strategy for skeletal diseases.

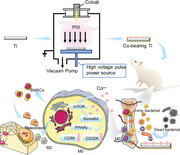

## 1 Introduction

Infected bone defects (IBDs) are debilitating disease that frequently arises during the care of complex open fractures or orthopedic operations, such as implantation.^[^ ^1^, ^2^, ^3^ ^]^ The World Health Organization (WHO) reports that there are roughly 1.5 million severe instances of bone infection worldwide each year, with the potential for amputations and, in certain situations, fatalities.^[^ ^4^ ^]^ *Staphylococcus aureus* (*S. aureus*) or other bacteria like *Escherichia coli* (*E. coli*) or *Pseudomonas aeruginosa* (*P. aeruginosa*) are the primary source of infections, which can have complex effects and hinder the process of bone regeneration.^[^ ^2^, ^5^, ^6^ ^]^

Traditional treatments for bone infection concentrate on preventing the formation of biofilms or eliminating planktonic bacteria. This involves the systemic delivery of broad‐spectrum antibiotics, such as sulfonamides, fluoroquinolones, or β‐lactams, for 4–6 weeks.^[^ ^7^, ^8^, ^9^ ^]^ However, the widespread use of antibiotics has many disadvantages, the most notable being the spread of drug resistance, which has been made worse recently by the development of COVID‐19.^[^ ^10^ ^]^ Biofilms formed by bacterial proliferation frequently obstruct antibiotic penetration, resulting in persistent infections that are challenging to treat.^[^ ^11^, ^12^ ^]^ Meanwhile, the prevalence of methicillin‐resistant *Staphylococcus aureus* (MRSA) is increasing, with infection rates reaching up to 50%, mainly due to the overuse of antibiotics.^[^ ^13^, ^14^, ^15^ ^]^

Another leading cause of delayed tissue healing is inflammation brought on by infections linked to biofilms.^[^ ^7^, ^16^, ^17^ ^]^ While unusually persistent inflammation can cause tissue damage, normal inflammation can eradicate bacterial infections and encourage tissue repair and regeneration through the action of inflammatory cells such as neutrophils and macrophages.^[^ ^18^, ^19^ ^]^ Indeed, a variety of toxins, including lipopolysaccharides (LPS), peptidoglycan, lipoproteins, and others, are released by pathogenic bacteria, and these cause macrophages to adopt a pro‐inflammatory (M1) phenotype.^[^ ^20^, ^21^, ^22^ ^]^ When macrophages maintain their M1 phenotype, many pro‐inflammatory molecules are released, exacerbating chronic inflammation in the surrounding tissue and preventing osteoblast production, interfering with bone integration and osteogenesis.^[^ ^23^, ^24^, ^25^ ^]^ Therefore, to address the serious issue of bacterial infections during bone defect restoration, the development of alternative, non‐antibiotic‐dependent therapies is urgently needed.

However, the surface modification procedures now in use do not meet the multifunctional needs of bone repair materials because their main goal is to maximize osteogenic capability.^[^ ^26^ ^]^ As a result, the best course of action for treating bone infections should be a combined approach that focuses on removing biofilms as effectively as possible while reducing localized inappropriate inflammation and encouraging bone integration.

Since inorganic elements are very stable and can facilitate doping and storage,^[^ ^3^ ^]^ metal ions are particularly relevant because they can induce numerous dying processes in bacteria,^[^ ^27^ ^]^ making it challenging to establish protective systems. However, excessive metal ions can cause cell damage or apoptosis, complicating the establishment of protective systems. The limited surface area of titanium implants can help regulate the concentration of metal ions, thereby achieving an antibacterial effect while minimizing harm to normal cells.^[^ ^28^ ^]^ For more than 40 years, cobalt has been a necessary trace element in implant alloys and is engaged in numerous critical biological processes.^[^ ^29^ ^]^ The effects of cobalt ions on the body vary depending on the dose. In particular, low dosages of cobalt ions stimulate tissue repair responses, but high doses show antibacterial properties without increasing the likelihood of resistance.^[^ ^30^, ^31^ ^]^ However, using cobalt ions in bone healing presents two complex problems. On the one hand, non‐physiological increases in cobalt ion concentration can be toxic and result in various harmful health effects, such as neurological, cardiovascular, and endocrine abnormalities, even though cobalt ions are a safe supplement.^[^ ^32^ ^]^ Therefore, controlling the release of cobalt ions is essential to lessen toxicity. Therefore, the results of several studies regarding cobalt's anti‐inflammatory properties are inconsistent. According to some research, cobalt doping in biomaterials can increase macrophages' pro‐inflammatory activity.^[^ ^33^, ^34^ ^]^ According to other research, cobalt supplementation encourages macrophages to polarize to the anti‐inflammatory (M2) type, which speeds up implant tissue regeneration.^[^ ^35^, ^36^ ^]^ These discrepancies could mainly result from variations in the experimental setups and cobalt element contents across different investigations. Therefore, controlling the material's cobalt element content is crucial for successful treatment outcomes in cases of bone infection. High adjustability (structure, composition, and thickness) and high adhesion strength are two benefits of the straightforward electrochemical surface modification method known as plasma immersion ion implantation (PIII) technology.^[^ ^37^, ^38^, ^39^ ^]^

**Figure 1:**
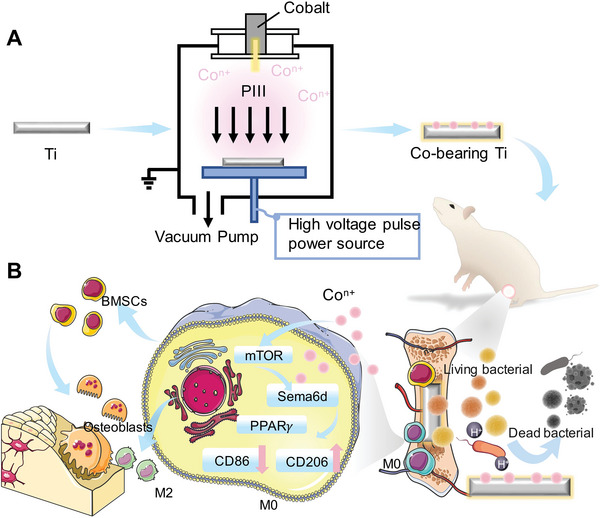
Schematic illustrating the Co‐bearing Ti design's complex system for boosting osteogenic activity and controlling immune responses to encourage the regeneration of diseased bone defects. A) Preparation processes of Co‐bearing Ti by Co PIII. B) The release of Co ions facilitates significant bioactivities, including promoting osteogenic differentiation of BMSCs, inducing immunoregulatory activity of macrophages (RAW264.7) toward M2 polarization, and antibacterial abilities.

In this work, we developed a multifunctional Co‐doped titanium implant using one‐step PIII technology for treating biofilm infections and promoting osteointegration (**Figure** **1**A). The Co content on the titanium surface is precisely regulated to enhance antibacterial properties while preserving optimal biocompatibility. Cobalt‐containing titanium implants have demonstrated outstanding efficacy in eradicating germs, decreasing inflammation, and stimulating bone regeneration in the infected bone defect model utilizing SD rats. A transcriptome analysis was conducted to identify potential molecular mechanisms underlying the enhanced anti‐inflammatory activity (Figure 1B). Results showed that cobalt ions deposited on titanium surfaces facilitate bone repair through the inhibition of inflammatory pathways, the induction of macrophage polarization to the M2 type, the secretion of associated inflammatory regulatory factors, and the promotion of osteoblast differentiation from bone marrow mesenchymal stem cells (BMSCs). Our work presents a promising approach for developing implant materials designed for long‐term treatment of bone defects. For the first time, the potential mechanism underlying the polarization of macrophages to the M2 phenotype induced by cobalt is elucidated from an inflammatory perspective, offering fresh theoretical and practical guidelines for developing materials for repairing bone infections.

## 2 Results

### 2.1 Scaffold Morphology and Surface Properties

**Figure 2:**
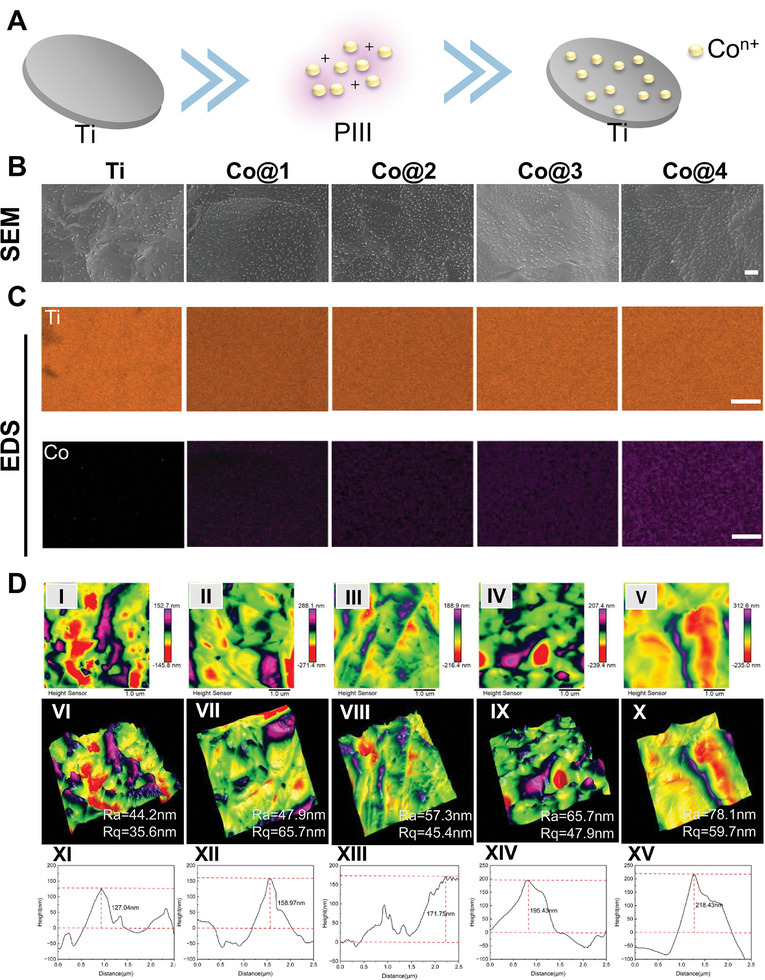
Design and surface morphology of Co‐bearing Ti. A) Schematic representation of the fabrication process of Co‐bearing Ti via Co‐PIII. B) SEM images depicting the surface morphology of the samples. Scale bar = 1 µm. C) Energy‐dispersive X‐ray spectroscopy (EDS) analysis confirms elements' uniform distribution across the samples. Scale bar = 2.5 µm. D) Atomic force microscopy (AFM) images presenting 2D surface topographies (I, II, III, IV, and V), 3D surface profiles (VI, VII, VIII, IX, and X), and depth profiles (XI, XII, XIII, XIV, and XV) of the samples. Scale bar = 1 µm.

The PIII technique incorporates Co elements into the titanium surface, as shown in **Figure** **2**A. Scanning electron microscopy (SEM) was used to describe the surface morphology of the samples. The results showed that the acid‐etched Ti samples had a flat surface with micro‐scale undulating morphology and that the surface topography was not considerably changed by Co ion implantation (Figure 2B).^[^ ^38^ ^]^ In this study, cobalt was utilized for its antimicrobial properties, immune modulation capabilities, and promotion of osteointegration. To achieve these objectives, ensuring the uniform distribution of cobalt within the titanium matrix is essential, which was confirmed by SEM elemental mapping. The cobalt (depicted in purple) was uniformly dispersed within the titanium matrix (shown in orange) (Figure 2C). Cobalt is likely to deposit on the titanium surface, potentially forming a stable cobalt‐containing oxide layer that becomes firmly integrated with the substrate during the PIII process.

In addition, the PIII samples' surface roughness was raised. The average roughness (Ra) and root mean square height (Rq) values for the corresponding samples were as follows in comparison to the PIII samples: The Ra values were 44.2 and 35.6 nm for Ti, 47.9 and 65.7 nm for Co@1, 57.3 and 45.4 nm for Co@2, 65.7 and 47.9 nm for Co@3, and 78.1 and 59.7 nm for Co@4. The Ra values of the Ti sample were the lowest, while the Co@1, Co@2, Co@3, and Co@4 samples showed an incremental increase in Ra values along with a corresponding rise in maximum fluctuation height. Ti < Co@1 < Co@2 < Co@3 < Co@4 is the sequence in which roughness increases (Figure 2D). The increased surface roughness increases the samples' specific surface area, and the right amount of roughness can help BMSCs adhere to surfaces and differentiate into osteoblasts.^[^ ^39^ ^]^

**Figure 3:**
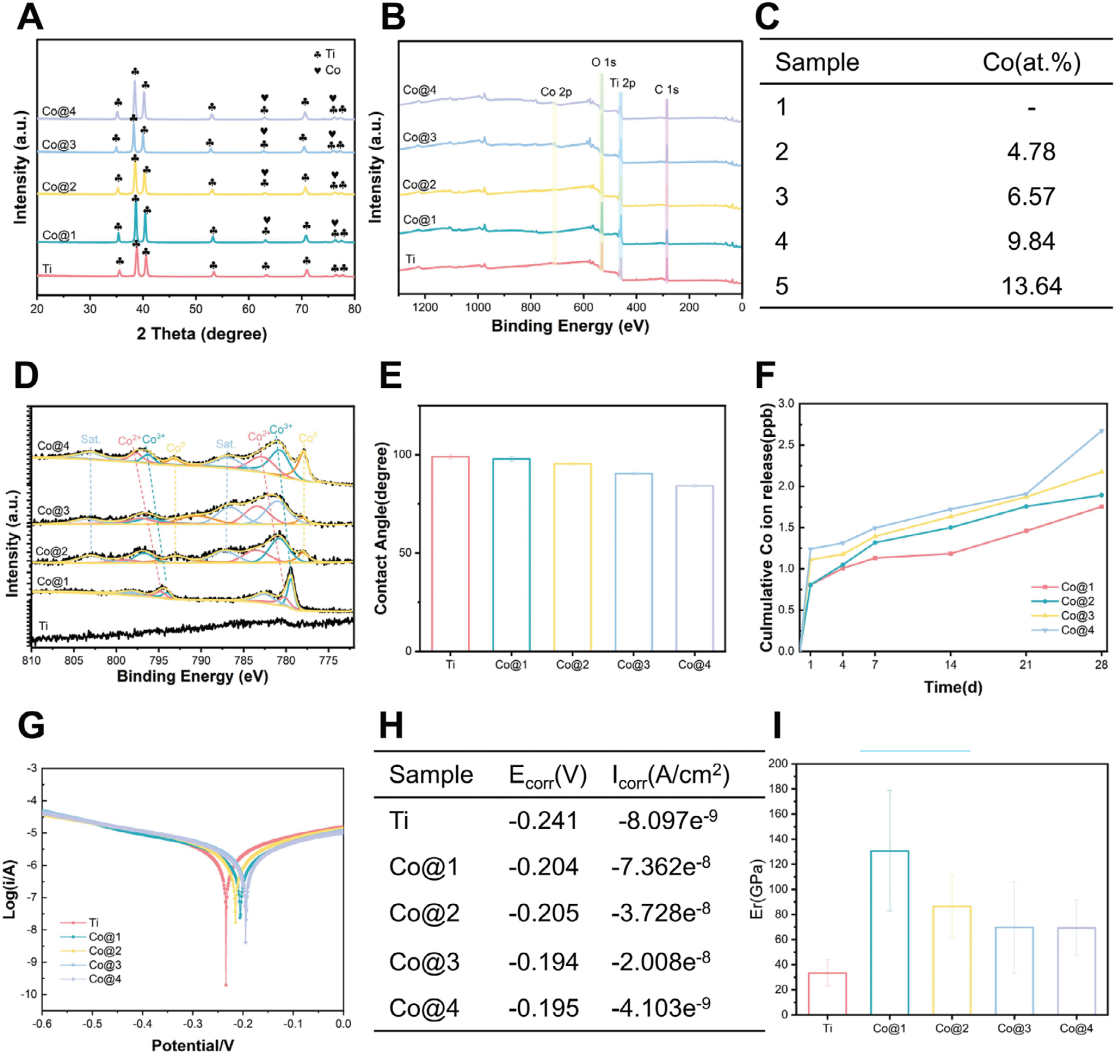
Comprehensive surface characterization of scaffold. A) XRD patterns illustrating the crystalline structure of the samples. B) XPS survey spectra provide an overview of the elemental composition on the surface. C) Quantitative elemental content determined from the XPS analysis. D) High‐resolution XPS spectrum of Co 2p region, detailing the oxidation states of cobalt on the sample surfaces. E) Water contact angles were measured to assess the hydrophilicity of the samples. F) Kinetic curves of cobalt ion release from the samples in PBS. G) Polarization curves derived from electrochemical tests indicate the corrosion resistance of the samples. H) Tabulated data corresponding to the polarization curves, presenting the electrochemical parameters. I) Elastic modulus of samples assessed by nanoindentation.

X‐ray diffraction (XRD) was used to describe the crystal structures of the materials (**Figure** **3**A). Anatase TiO_2_ was found to be the main phase in the Ti samples. Co^2+^ may enter interstitial lattice sites or occupy substitutional sites by placing Ti^4+^ in the host lattice following Co‐PIII. This makes sense since, for hexacoordinated centers with octahedral geometry, Co^2+^ and Ti^4+^ have ionic radii that are pretty similar—79 and 74.5 pm, respectively.^[^ ^33^ ^]^ Nevertheless, no appreciable variations were seen in the samples' XRD patterns. As a result, the surface chemical characteristics were determined using X‐ray photoelectron spectroscopy (XPS), a surface‐sensitive characterization technique. Figure 3B,C show the samples' quantitative elemental composition and XPS survey spectra. Peaks attributed to cobalt were found on the surfaces of the Co@1, Co@2, Co@3, and Co@4 samples, with cobalt concentrations of 4.78, 6.57, 9.84, and 13.64%, respectively, in comparison to the Ti sample. This suggests that the Co content in the samples can be improved by administering a higher dose of cobalt ion implantation. Figure 3D displays the Co 2p high‐resolution XPS spectra. While peaks at 781.1 and 797.0 eV, along with two satellite peaks at 785.3 and 801.9 eV, are associated with Co^2+^ 2p3/2 and Co^2+^ 2p1/2, suggesting that some Co^2+^ ions in the material are partially oxidized to Co^3+^ ions, peaks centered at 778.7 and 794.0 eV are assignable to Co^3+^ 2p3/2 and Co^3+^ 2p1/2.^[^ ^40^, ^41^ ^]^ Notably, the 778.3 and 793.0 eV peaks correspond to Co^0^ 2p3/2 and Co^0^ 2p1/2.^[^ ^42^ ^]^

Since hydrophilicity affects early cell adhesion, spreading, and proliferation, it is a crucial property of biomedical scaffolds.^[^ ^43^ ^]^ The contact angles for the Ti, Co@1, Co@2, Co@3, and Co@4 samples are 99.00°, 97.89°, 95.36°, 90.43°, and 84.24°, respectively. Figure 3E shows the wettability of the samples. After PIII treatment, the samples' hydrophilicity increased along with their surface Co content. Co nanoparticles were deposited on the scaffold surface, roughening it up and reducing the contact angle on the modified surface.^[^ ^37^ ^]^ The metal ion release from PIII samples in PBS during 28 days was assessed using ICP‐MS, with the ion concentrations released by the PIII samples displayed in Figure 3F. This was done since large quantities of metal ions have the potential to be cytotoxic.^[^ ^44^ ^]^ PBS was chosen over H_2_O and saline solutions as it resembles the physiological environment more closely. The concentration of implanted ions increased gradually with extended soaking time, with the maximum cobalt ion release in the PBS solution being 2.672 ppb for all samples, which is also significantly lower than the safe concentration of cobalt ions in the human body.^[^ ^30^ ^]^ Concurrently, the introduction of different Co elements affected the electrochemical properties of the sample surfaces. Figure 3G,H exhibits the polarization curves of the samples in physiological saline together with the related data. As Co nanoparticles with a greater corrosion potential were uniformly spread on the Ti matrix following Co‐PIII, the changed samples' corrosion potential (E_corr_) shifted positively compared to the Ti sample. However, the corrosion current (I_corr_) of the modified samples was higher than that of the Ti sample, indicating that once a corrosion reaction occurs, it accelerates rapidly. Co@Ti with varying Co concentrations exhibit an initial increase followed by a decrease in their elastic modulus, which is consistent with the trend seen in other doped alloys.^[^ ^45^ ^]^ The elastic modulus of all cobalt‐ion‐implanted alloys, as illustrated in Figure 3I, is greater than that of pure Ti (33.40 GPa), with Co@1 exhibiting the highest modulus (130.56 GPa). Cell growth will be aided by an elastic modulus that is suitable.^[^ ^46^ ^]^

### 2.2 Scaffold Biocompatibility

The materials were seeded with several cell lines (BMSCs, RAW264.7) to examine the impact of cobalt ion implantation on the viability and proliferation of the cells. According to the CCK‐8 assay, on days 1, 4, and 7, RAW264.7 and BMSCs showed increased proliferative activity on the ion‐implanted samples (Figure S1A,C, Supporting Information). Most of the seeded cells remained viable and adhered to the material surface after 1, 4, and 7 days of culture, which aligns with the trends shown with the live/dead staining and the CCK‐8 results (Figure S1B,D, Supporting Information). According to these findings, materials with cobalt ion implantation appear to be more biocompatible than those with only pure Ti. PIII treatment increases surface roughness and hydrophilicity, which aids in enhancing cell adhesion and proliferation.^[^ ^47^ ^]^ Moreover, favorable results have been obtained from the clinical usage of cobalt‐based alloys in hip and knee replacements, and their safety has been successfully confirmed in vivo.^[^ ^48^, ^49^ ^]^ Compared to applying bioactive compounds or nano‐modifications on titanium surfaces, we think using Co‐doped elements offers more consistent biosafety and potential for clinical translation. The Co@2 group had the fastest rate of proliferation for BMSCs. On RAW264.7 cells, however, the Co@3 group showed the most substantial proliferative effect. Consequently, the Co@2 and Co@3 groups were chosen to finish the ensuing studies.

### 2.3 Antibacterial Efficacy of Scaffold In Vitro

**Figure 4:**
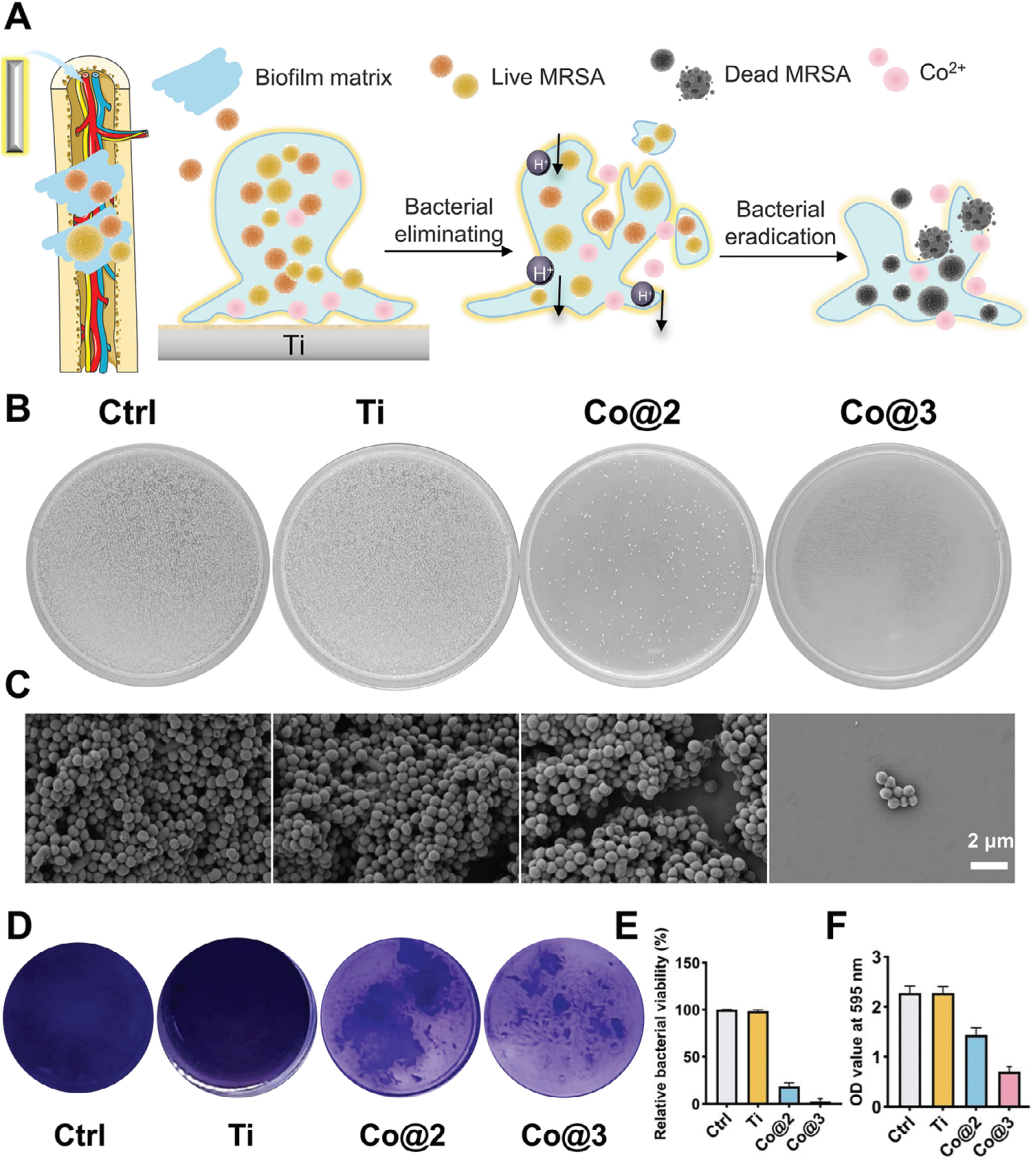
Co‐Ti effectively eliminates MRSA biofilm. A) Schematic diagram of the antibiofilm mechanism of Co‐Ti, illustrating the transition from biofilm matrix disruption to bacterial elimination and eradication. B) MRSA colonies visualized on agar plates after treatment with different samples: Control, Ti, Co@2, and Co@3.C) SEM images of MRSA biofilms following incubation with different samples, demonstrating morphological changes and bacterial removal. Scale bar = 2 µm. D) Crystal violet staining of biofilm biomass after treatment with Control, Ti, Co@2, and Co@3, showing reduced biofilm formation in Co@3‐treated samples. E) Relative bacterial viability of MRSA after treatment with various samples, quantified as a percentage. F) Quantification of biofilm biomass at OD 595 nm after treatment with different samples, with results shown as mean ± SD (*n* = 3).

Persistent bacterial infections in osseous defects can have severe consequences, including marked inflammation, localized osteolysis, and vascular compromise. Rapid bacterial eradication is crucial for restoring the disturbed skeletal immunological niche and improving treatment outcomes.^[^ ^50^ ^]^ The antibacterial efficacy of the Co‐Ti scaffold against MRSA biofilm formation was evaluated using both quantitative and qualitative assays. The schematic illustration of the antibiofilm mechanism (**Figure** **4**A) demonstrates that the Co ions released from the Co‐Ti scaffold can effectively penetrate the biofilm matrix, resulting in the eradication of MRSA by disrupting bacterial metabolic activity and membrane integrity.

To investigate the impact of Co‐Ti on MRSA biofilm formation, colony‐forming units (CFUs) on agar plates were analyzed following exposure to different samples (Figure 4B). The Co@3‐treated group exhibited a substantial reduction in bacterial colonies compared to both the control and Ti groups, indicating a potent antibacterial effect of the scaffold. SEM further confirmed these findings, revealing a pronounced disruption of the MRSA biofilm structure with a significant decrease in bacterial density upon treatment with Co@3 (Figure 4C). In contrast, the Ti‐treated group showed minimal impact on biofilm integrity.

Biofilm biomass was assessed using crystal violet staining, and a marked reduction was observed for the Co@3‐treated samples compared to the control and Ti groups (Figure 4D). The quantification of relative bacterial viability (Figure 4E) demonstrated that Co@3 treatment led to a significant decrease in viable MRSA cells, with viability levels falling below 30%, underscoring the efficacy of cobalt modification in enhancing the scaffold's antibacterial properties. Moreover, the optical density (OD) values at 595 nm (Figure 4F) further corroborated these results, indicating a considerable decline in biofilm biomass following Co@3 treatment.

To further evaluate the time‐dependent antibiofilm effects of the Co@3 scaffold, biofilm biomass was monitored at 2, 4, and 6 h post‐treatment (Figure S2, Supporting Information). A substantial reduction in biofilm coverage was evident as early as 2 h after treatment, with progressive decreases at subsequent time points (Figure S2A, Supporting Information). Quantitative analysis of the biofilm mass confirmed this trend, with a significant reduction in OD values observed over time (Figure S2B, Supporting Information). These results demonstrate the rapid and sustained antibiofilm activity of Co@3 against MRSA.

Overall, these findings highlight the robust antibacterial and antibiofilm capabilities of the Co‐modified Ti scaffold, making it a promising candidate for preventing bacterial infections in biomedical applications. The antibacterial impact of Co‐Ti is attributed to the release of Co^2+^ ions and the micro‐galvanic effect. Specifically, it is likely that the associated micro‐galvanic activity disrupts bacterial respiration and energy metabolism, leading to cellular damage and ultimately, bacterial death, at the same time cobalt ions may interfere with bacterial enzyme systems and cell wall synthesis, thereby inhibiting their growth.^[^ ^33^, ^37^, ^51^, ^52^ ^]^ As illustrated in Figure 4A, this dual mechanism of action suggests that cobalt ions play a significant role in both directly impairing bacterial metabolism and indirectly disrupting essential cellular processes, which together contribute to the observed antimicrobial effects.

### 2.4 Scaffold for Modulating RAW 264.7 Macrophages

**Figure 5:**
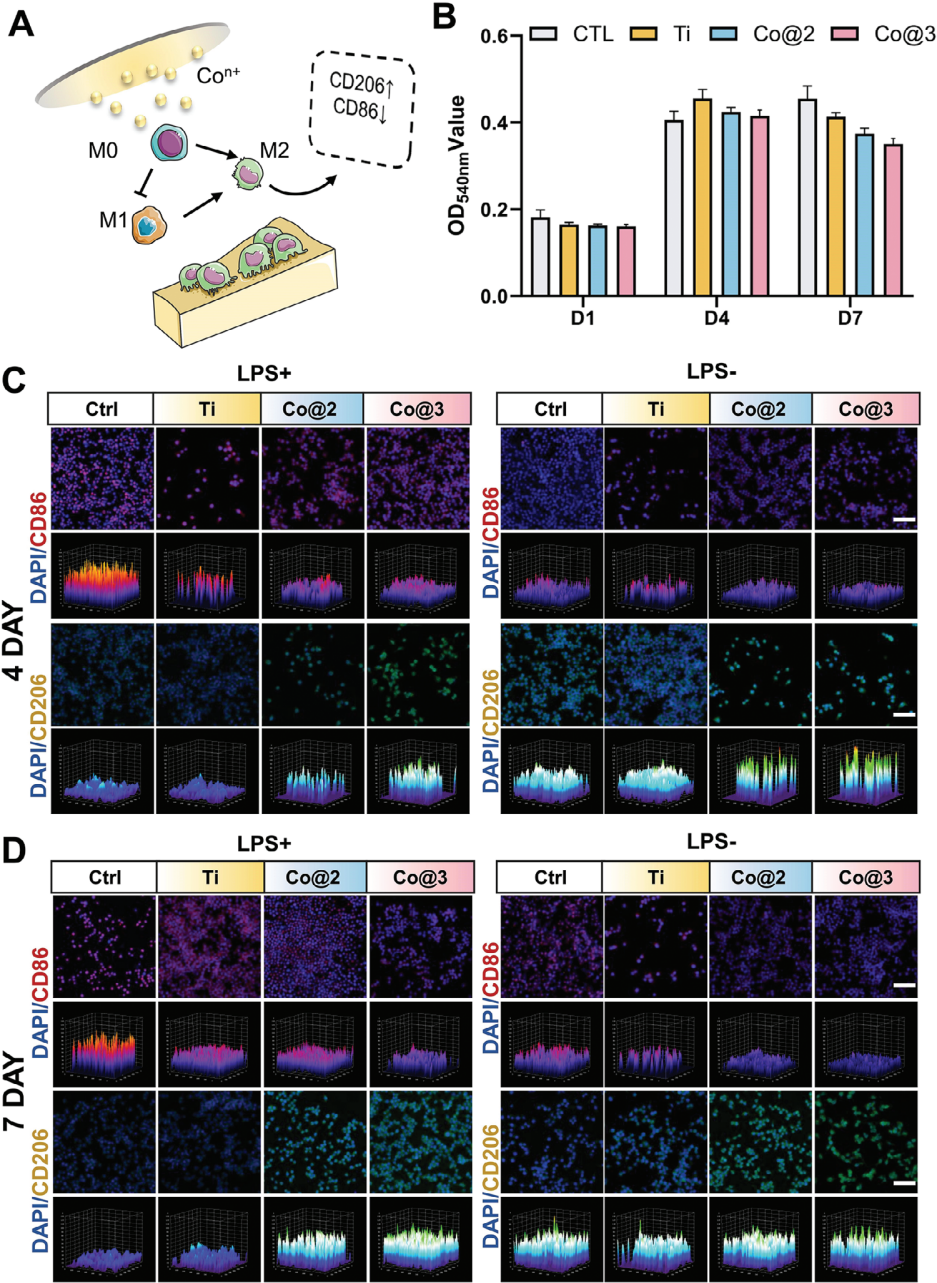
In vitro macrophage polarization on various samples. A) Schematic representation of the immunomodulatory activity of the samples. B) Nitric oxide (NO) content measured using the Griess reagent (*n* = 4). C) Fluorescence microscopy images showing CD86 and CD206 staining of RAW264.7 cells cultured with the samples on day 4. D) Corresponding fluorescence microscopy images of CD86 and CD206 staining for RAW264.7 cells cultured with the samples on day 7. Scale bar = 50µm.

Innate immune cells called macrophages are essential for the antimicrobial immune response and the immunoregulation of bone defect healing.^[^ ^53^ ^]^ Bacterial biofilms and biomaterials in bone infections can bring on a “frustrated” state in macrophages. This can result in a marked decrease in antibacterial efficacy and a postponement of the M1‐M2 transition, which can cause chronic inflammation and inflammatory reactions.^[^ ^54^ ^]^ The released Co^2+^ from the samples is anticipated to trigger an anti‐inflammatory response, reducing excessive inflammation and producing a suitable immunological reaction to pertinent literature reports^[^ ^49^ ^]^ (**Figure** **5**A). To investigate the anti‐inflammatory effects of the material after cobalt ion implantation, LPS stimulation was used to simulate a chronic inflammatory environment, and the activation of the inflammatory response was determined by observing with an optical microscope and measuring the NO content in the supernatant (Figure S3A,B, Supporting Information). LPS activates the inflammatory response in RAW264.7 cells, leading to a significant enhancement in the secretion of NO in the supernatant following LPS stimulation. Moreover, the inflammatory response induced by LPS gradually intensifies over time, while the material with cobalt ion implantation more effectively inhibits LPS‐induced NO production (Figure 5B). Immunofluorescence labeling for the M1 and M2 phenotypic markers was used to evaluate the immunomodulatory qualities of the samples on RAW264.7. For immunofluorescence staining assays, two distinct macrophage populations—CD206 (green; M2 macrophages) and CD86 (red; M1 macrophages)—were used to detect the polarization of macrophages on the sample surfaces (Figure 5C,D). Treatment groups suppressed M1 marker CD86 expression while simultaneously promoting M2 marker (CD206) expression, regardless of whether macrophages were stimulated with LPS. In the presence of inflammation, on day 4, the expression of CD86 in the treatment group was lower than that in the LPS and Ti groups (Figure 5C). Additionally, on day 7, the expression of CD86 in the treatment group decreased, while the expression of CD206 increased (Figure 5D). Additionally, quantitative analysis of immunofluorescence results further revealed that the treated samples could induce macrophages to polarize from the M1 phenotype to the M2 phenotype, with the induction effect enhancing as the cobalt content in the samples increased (Figure S4, Supporting Information). The findings demonstrate that the Co@2 and Co@3 groups exhibit strong immunomodulatory action. By causing M2 macrophage polarization, the release of Co^2+^ can facilitate immunological modulation and tissue remodeling.

### 2.5 Mechanistic Analysis of Sample‐Regulated Macrophage Polarization

**Figure 6:**
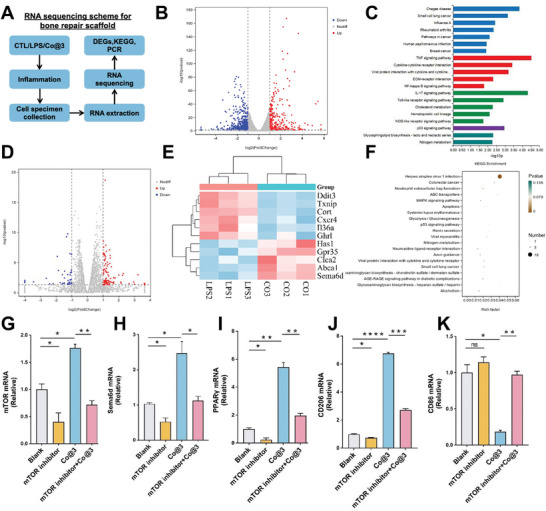
Transcriptomic Profiling of Scaffold Effects. A) Overview of the RNA sequencing strategy for analyzing the impact of the bone repair scaffold. B) Volcano plot displaying upregulated (red) and downregulated (blue) DEGs. C) GO analysis categorizes DEGs by cellular component, process, and function. D) DEGs volcano plot comparing Co‐treated to LPS conditions. E) Heatmap of the 11 most significantly regulated genes. F) KEGG pathway analysis indicating enriched pathways. G–K) Expression of genes mTOR, Sema6d, PPARγ, CD206, and CD86 on day 4. (*n* = 3, ns means not significant. *p < 0.05, **p < 0.01, ***p < 0.001, and ****p < 0.0001).

To clarify the impact of cobalt‐containing implants on the functional regulation of macrophages under infectious conditions, we conducted RNA sequencing analysis on macrophages co‐cultured with Co@3 in the presence of LPS (**Figure** **6**A). The volcano plot in Figure 6B demonstrates that the LPS group had 222 genes highly elevated and 230 genes significantly downregulated compared to the control group CTL. This implies significant changes in gene expression. Furthermore, a Gene Ontology (GO) analysis was conducted, wherein cellular components are indicated in red, biological processes are shown in blue, molecular functions are displayed in green, and metabolism is shown in purple (Figure 6C). Upon the introduction of LPS, several inflammatory pathways were triggered, such as IL‐17, TNF‐α, and toll‐like pathways, in contrast to the control group. The validation of these pathways in many investigations confirmed the successful creation of the LPS‐induced inflammatory model, which facilitates macrophage polarization toward the M1 phenotype.^[^ ^55^ ^]^ Additionally, comparing the differently expressed genes (DEGs) between the Co@3 and LPS groups showed substantial differences, with 40 downregulated genes and 97 upregulated genes (Figure 6D). The heatmap illustrates the fold changes in gene expression between the Co@3 and LPS groups, representing differentially expressed genes. The Co@3 group exhibited a significant increase in the expression levels of anti‐inflammatory related genes (Has1, Abca1etc.) and a significant decrease in the expression levels of pro‐inflammatory related genes (Txnip, Cxcr4, Il36a, etc.) (Figure 6E). All differentially expressed genes underwent GO analysis, which showed enrichment in immune response regulation, cytoskeleton organization, cell adhesion, and proliferation. Implants containing cobalt facilitate these processes, which, by morphological and polarized growth, may induce M2 polarization. The KEGG pathway enrichment analysis reveals alterations in the pathways associated with macrophage polarization, including TNF formation, MAPK signaling, apoptosis, and p53 signaling pathways. These findings imply that the material under consideration has the potential to decrease the generation of pro‐inflammatory cytokines, improve the elimination of apoptotic cells, and mitigate chronic inflammatory conditions (Figure 6F).

After reviewing the literature,^[^ ^56^ ^]^ it is known that the mTOR‐Sema6d‐PPARγ signaling axis mediates the polarization of M2 macrophages and that Sema6d overexpression can facilitate this process. The Co@3 group showed significant expression of Sema6d in the sequencing results of this investigation. Thus, we hypothesize that this pathway may impact the mechanism that promotes M2 polarization. As a result, we used Q‐PCR to confirm the alterations in the expression of pertinent essential genes. The findings demonstrated that whereas CD86 expression declined, mTOR, Sema6d, PPARγ, and CD206 expression rose in the Co@3 group. Adding a mTOR inhibitor resulted in a rise in CD86 expression and a decrease in mTOR, Sema6d, PPARγ, and CD206 expression. This suggests Co@3 might encourage M2 macrophage polarization by activating the mTOR‐Sema6d‐PPARγ pathway. The identification of this mechanism offers further molecular proof that the scaffold stimulates macrophage polarization.

We provide a molecular explanation: by altering the immunological milieu and stimulating osteogenesis, cobalt‐containing implants aid in healing inflammatory bone defects. Following Co@3 implantation, the release of Co^2+^ triggers macrophage migration to the implant site, where they polarize into M2 macrophages, actively contribute to the remodeling of the immunological milieu, and encourage the production of new bone. Ultimately, this results in healing bone abnormalities caused by inflammation, returning them to a state akin to normal bone tissue.

### 2.6 The Impact of Samples on the Osteogenic Differentiation of BMSCs and the Inhibition of Osteoclast Activation

**Figure 7:**
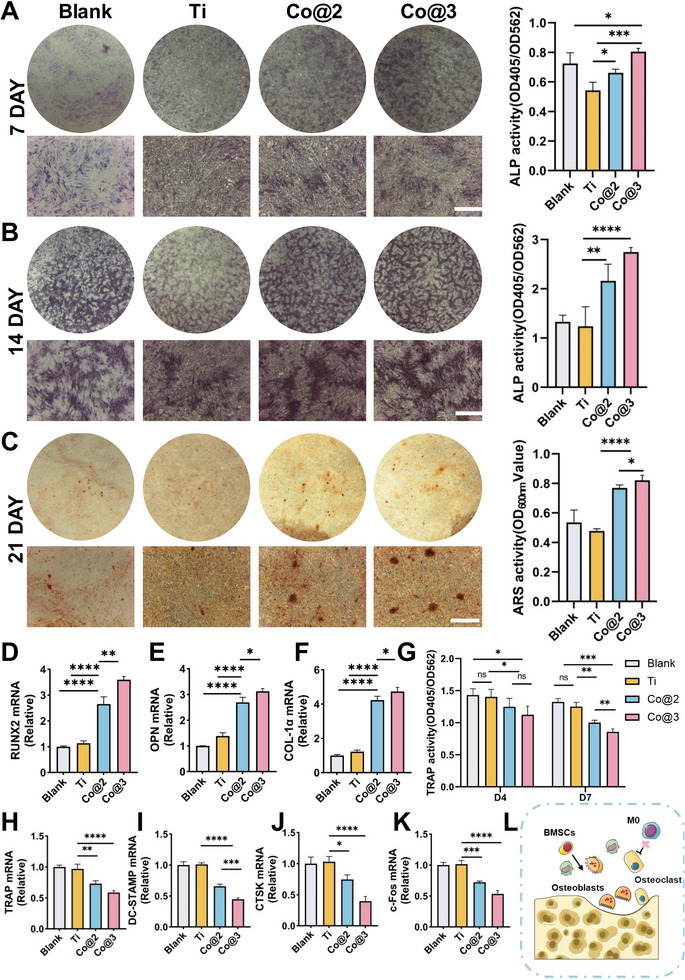
Osteogenesis and osteoclast on various samples in vitro. A) ALP staining and quantitative activity analysis on day 7. B) ALP staining and activity quantification on day 14, expressed as OD_405_/OD_562_. C) Alizarin Red staining for calcium mineralization on day 21, expressed as OD_600_. D–F) Expression of osteogenic genes RUNX‐2, COL‐1α, OPN in BMSCs on day 7. G) Tartrate‐resistant acid phosphatase (TRAP) activity in RAW264.7 cells on days 4 and 7, expressed as OD_405_/OD_562_. Scale bar = 200 µm. H–K) Expression of osteoclastogenic genes TRAP, DC‐STAMP, CTSK, and c‐Fos in RAW264.7 cells on day 4. L) Schematic of the mechanisms of osteoclast and osteoblast activity induced by Co‐bearing Ti (*n* = 3). (ns means not significant. *p < 0.05, **p < 0.01, ***p < 0.001, and ****p < 0.0001).

The initial goal was to create and evaluate an osteogenic titanium implant that was enhanced with cobalt, taking advantage of this material's special bioactivity to encourage bone integration and healing. This was part of the multi‐stage development process for the finished product. Alkaline phosphatase (ALP) is a valuable marker in the early stages of osteogenic differentiation. The ALP activity of BMSCs cultivated with the treated materials for 7 and 14 days rose significantly compared to the Ti group, particularly in Co@3, a result in line with the quantitative detection of ALP (**Figure** **7**A,B). Alizarin Red staining was also used to assess mineralization during the latter phases of osteogenic differentiation. According to the results displayed in Figure 7C, the treated groups had significantly increased matrix mineralization on day 21. The following implies that Co@3 significantly affects the proliferation and osteogenic differentiation of BMSCs, which aligns with the ALP and cell viability tests. Further investigation was conducted into how the samples affected the expression of genes linked to osteogenic development in BMSCs. The gene expression levels of runt‐related transcription factor (Runx2), type I collagen (COL‐1α), and compared to Ti, osteopontin (OPN) was elevated by cobalt inclusion, as shown in Figure 7D–F. Cobalt‐containing samples may promote osteogenic transcription factors such as Runx2, stimulate collagen synthesis, and modulate osteopontin levels, thereby facilitating the differentiation of osteoblasts and the formation of bone matrix.^[^ ^49^, ^57^, ^58^, ^59^, ^60^ ^]^

According to reports, the production of osteoclasts can be inhibited by cobalt ions at specific doses.^[^ ^61^ ^]^ Consequently, we assessed the impact of cobalt‐containing samples on RAW264.7 osteoclast activity over two‐time points, the 4th and 7th day of culture, within an inflammatory environment. The results from TRAP quantitative detection data (Figure 7G) demonstrated that samples incorporating cobalt led to a significant reduction in osteoclast activity on both days, suggesting a sustained suppressive effect on osteoclastogenesis. To evaluate the impact of various samples on osteoclast function, the expression of osteoclast differentiation‐related genes, such as TRAP, DC‐STAMP, CTSK, and c‐Fos was examined.^[^ ^62^ ^]^ The results indicated that cobalt‐containing samples suppress the expression of osteoclast‐related genes (Figure 7H–K). These discoveries suggest that cobalt‐containing implants may aid in the healing of bone defects by stimulating BMSCs' osteogenic differentiation and inhibiting osteoclast activity (Figure 7L). These findings support earlier research by Sun,^[^ ^31^ ^]^ which indicates that Co^2+^ aids in the repair of bone tissue.

### 2.7 In Vivo Therapeutic Performance of the Samples

**Figure 8:**
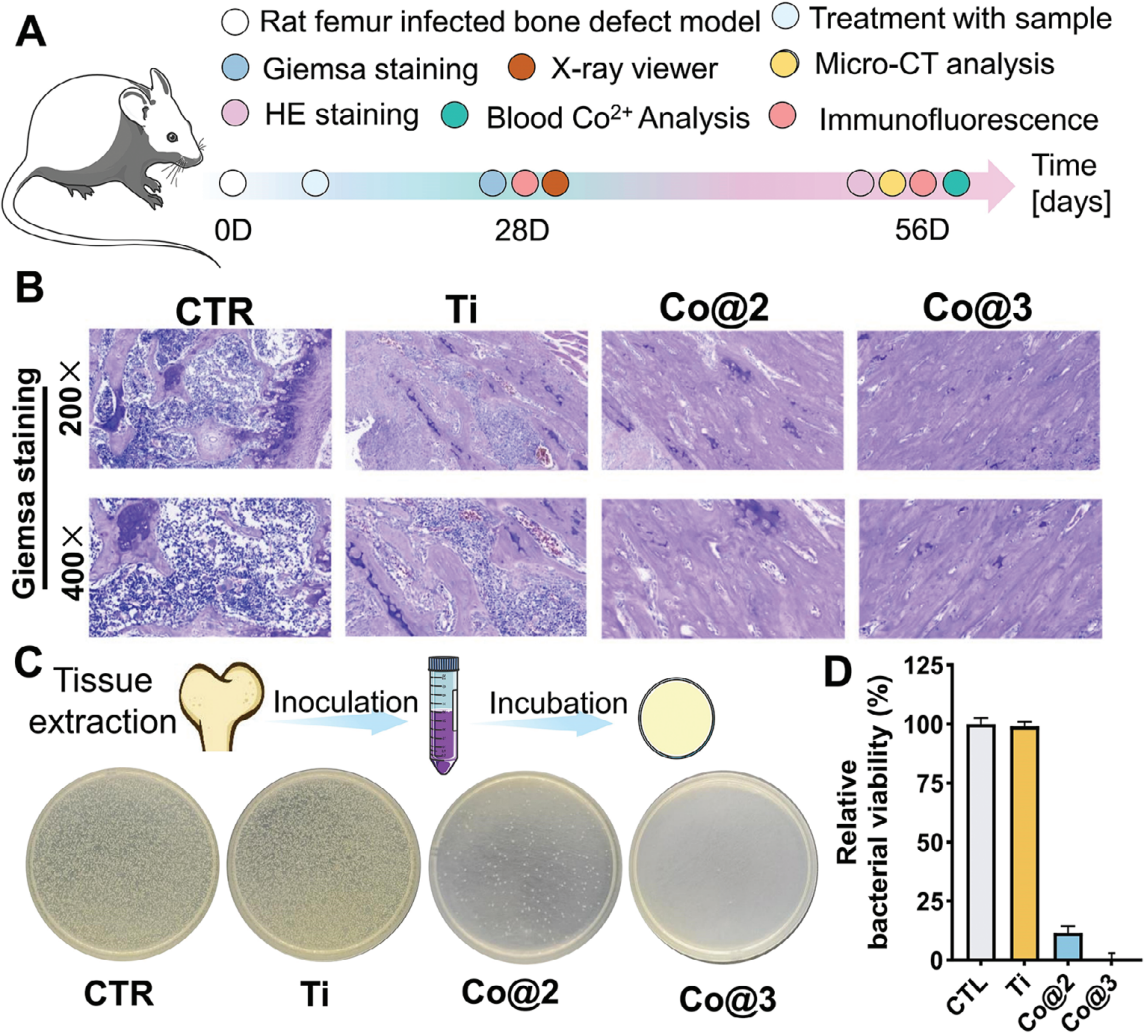
Comprehensive analysis of in vivo antibacterial activity. A) Timeline schematic of the in vivo study protocol. B) Giemsa staining of tissue sections to evaluate bacterial infection. C) Schematic diagram and photographic representation illustrating tissue sampling and subsequent bacterial culture for comparative analysis among the groups. D) Quantitative assessment of antibacterial efficacy, determined by colony plate counts following sample incubation (*n* = 4).

Promising in vitro bactericidal, immunomodulatory, and osteogenic activities have been demonstrated by cobalt‐containing implants. In contrast to in vitro settings, the interactions between bacteria and host cells and between cells are more complex in an actual in vivo implant environment. We created an infected bone defect model in the rat femur. We implanted the materials to investigate their possible impacts and mechanisms to assess the therapeutic efficacy of cobalt‐containing implants in vivo (**Figure** **8**A). Before evaluating the therapeutic effects, we collected the main organs for pathological staining investigation, including the liver, spleen, heart, lungs, and kidneys, to test the materials' biocompatibility. The tissue morphology was similar in all groups, with no anomalies, as shown in Figure S5 (Supporting Information), suggesting that the implants have excellent biocompatibility. To check for Co^2+^ levels, we also took rat blood samples. The Co@2 and Co@3 groups' levels of Co^2+^ did not significantly rise compared to the control group, indicating that the samples' Co^2+^ did not permeate the rat's body and proving the material's safety and dependability (Figure S6, Supporting Information).

Subsequently, the therapeutic efficacy for IBDs infected bone defects was assessed. Giemsa‐stained sections revealed a higher presence of *Staphylococcus aureus* in the CTL and Ti groups, which lacked effective antibacterial capabilities. In contrast, the Co@2 and Co@3 groups demonstrated efficient elimination of *S. aureus* due to the release of Co^2+^, underscoring their robust antibacterial activity against implanted bacteria (Figure 8B). Surrounding tissues from the defects were collected for bacterial culture analysis (Figure 8C,D). The findings indicated a marked decrease in bacterial presence in tissues adjacent to the cobalt‐containing titanium implants, whereas the control and standard titanium groups exhibited substantial bacterial colonies. These in vivo results highlight the sustained antibacterial properties of the cobalt‐containing titanium implants, aligning with their potential to mitigate the risk of post‐surgical infections and foster a sterile environment conducive to healing.

**Figure 9:**
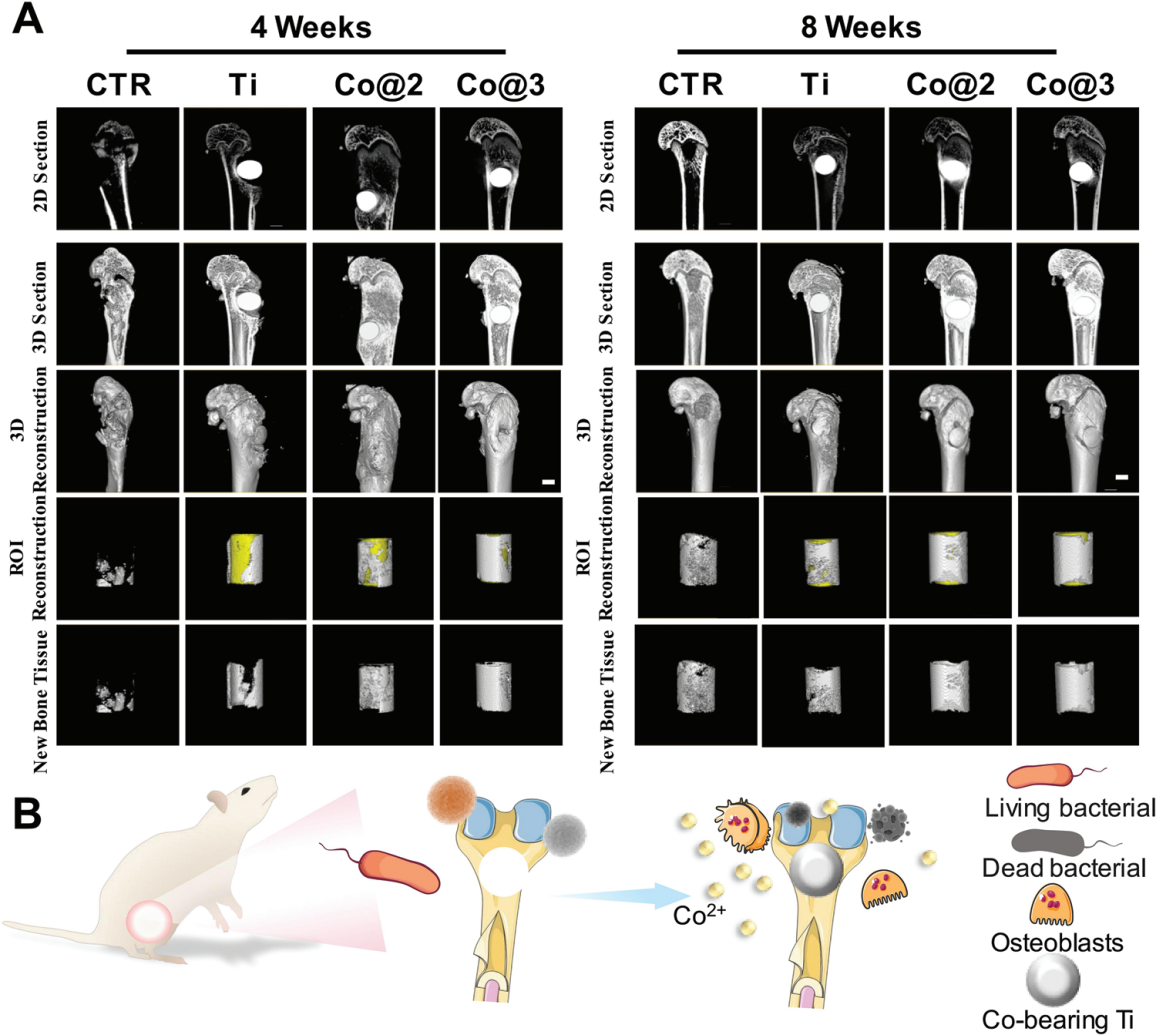
In vivo bone regeneration assessment. A) Micro‐CT imaging of the tibial defect from coronal, sagittal, and transverse perspectives. Scale bar = 2 mm. B) Schematic illustration of antibacterial and osteogenic activities of cobalt‐containing implants.

To further assess the formation of new bone in vivo following the implantation of the composite scaffold, micro‐CT scans were performed on the proximal end of the right tibia in rats, with 3D reconstructions carried out at 4 and 8 weeks (**Figure** **9**A). A good healing response in the bone around the cobalt‐containing implant groups was indicated by the 2D sections taken from Micro‐CT scans.

Bone tissue has penetrated the internal structures of Co@2 and Co@3, as seen in the reconstructed images of freshly formed bone. This suggests that the bone in the cobalt‐containing implant groups has healed well. As time extended to 8 weeks, the Co@3 group contained more organized and enhanced regenerative bone tissue, and maintained its structural integrity without local fractures. The quantity and maturity of newly formed bone in the Co@2 and Co@3 implants are more visually satisfying, implying that they improve the in vivo osteogenic capacity.

**Figure 10:**
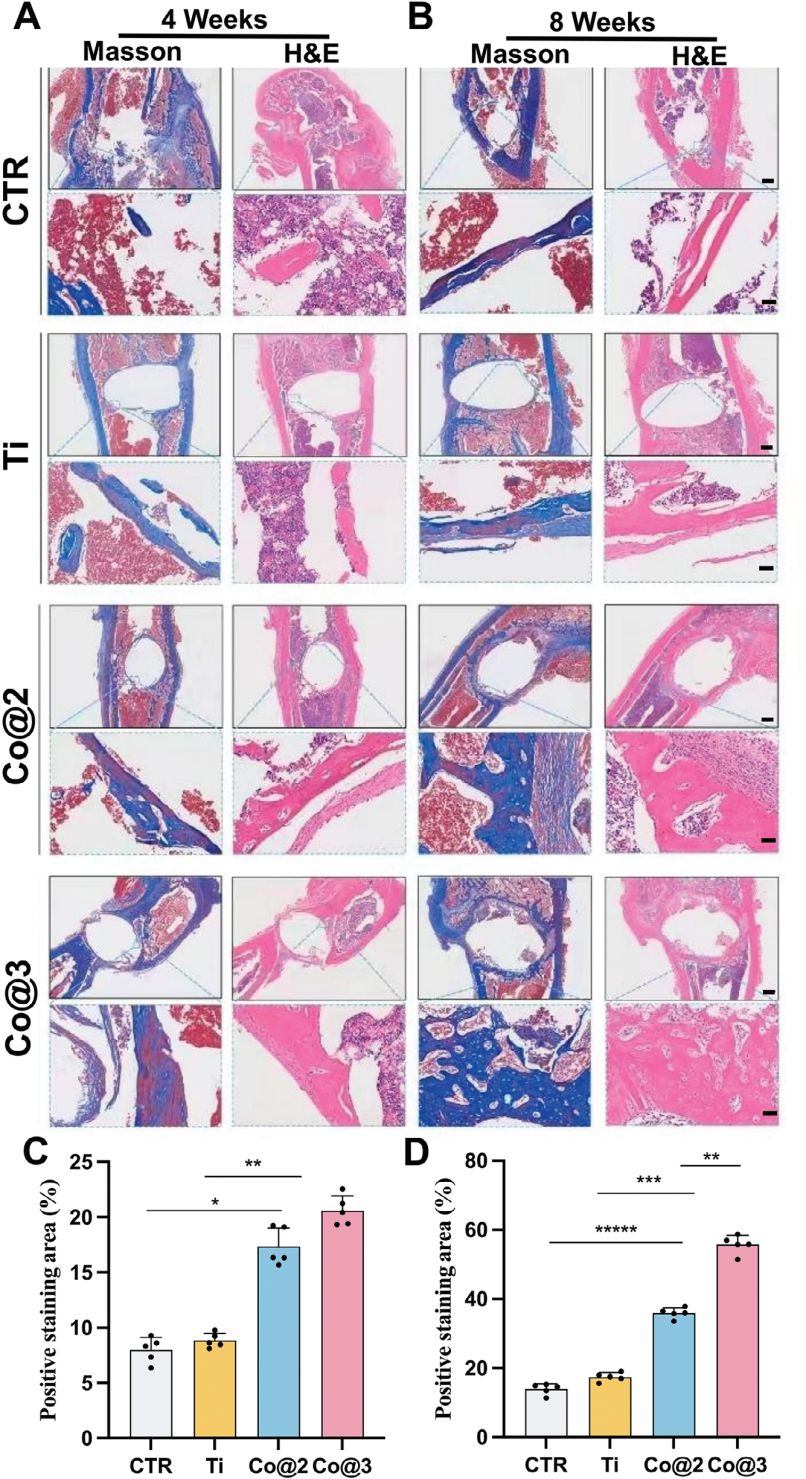
Histological assessment of implanted samples at weeks 4 and 8. A) H&E and Masson's trichrome staining at 4 weeks to evaluate infection and bone regeneration. B) Corresponding histological evaluation at 8 weeks demonstrating advanced healing. Scar bar: 200 and 50 µm. C,D) Quantitative analysis of Masson's staining reflecting bone formation at weeks 4 and 8, respectively (*n* = 5). (*p < 0.05,**p < 0.01, ***p < 0.001, and ****p < 0.0001).

A vital component of a successful implant placement is osseointegration at the bone‐implant interface. Hematoxylin and Eosin (H&E) and Masson's Trichrome staining are used in histological evaluations of newly formed bone surrounding implants to measure the quality of bone integration.^[^ ^7^ ^]^ Both the control and Ti scaffold groups had cavities after implantation for 4 weeks, as shown by H&E staining, with the borders of the bone defects not attached to the implants (**Figure** **10**A). By comparison, the Co@2 and Co@3 groups showed a much higher proportion of new bone (blue staining, Figure 10A,C) than the Ti group. The majority of the new bone was well‐mineralized mature trabecular bone. In contrast, the control group showed minimal mature bone formation. In the 8th week, the trends observed were similar to those of the 4th week. With the implant‐bone defect edges attached to the connective fibrous tissue, H&E staining demonstrated the creation of significant new bone tissue in the Co@2 and Co@3 groups, suggesting continued osseointegration and growth at the host‐implant interface (Figure 10B). The increased osteogenic activity was further supported by Masson's trichrome staining, which showed that the Co@2 and Co@3 groups had higher percentages of newly formed bone, as indicated by more extensive blue staining, which is a sign of mature bone tissue (Figure 10B). More specifically, the area of new bone accounted for 55.73% of the Co@3 group, much more than the 17.39% shown in the Ti group (Figure 10D). These findings validate cobalt‐containing implants' effectiveness in promoting bone growth and combating foreign bacteria at the location of diseased bone defects (Figure 9B).

### 2.8 The In Vivo Mechanism of Action of the Sample in the Treatment of Bone Infection

In investigating the potential of cobalt‐containing implants to repair bone infection defects through immunomodulation, this study employed immunofluorescence staining techniques to analyze rat femur tissue, aiming to reveal their comprehensive effects in promoting osteointegration and repairing infectious bone defects. It is well‐established that excessive inflammatory responses can inhibit the osteogenic process, leading to delayed bone healing or even implant failure.

**Figure 11:**
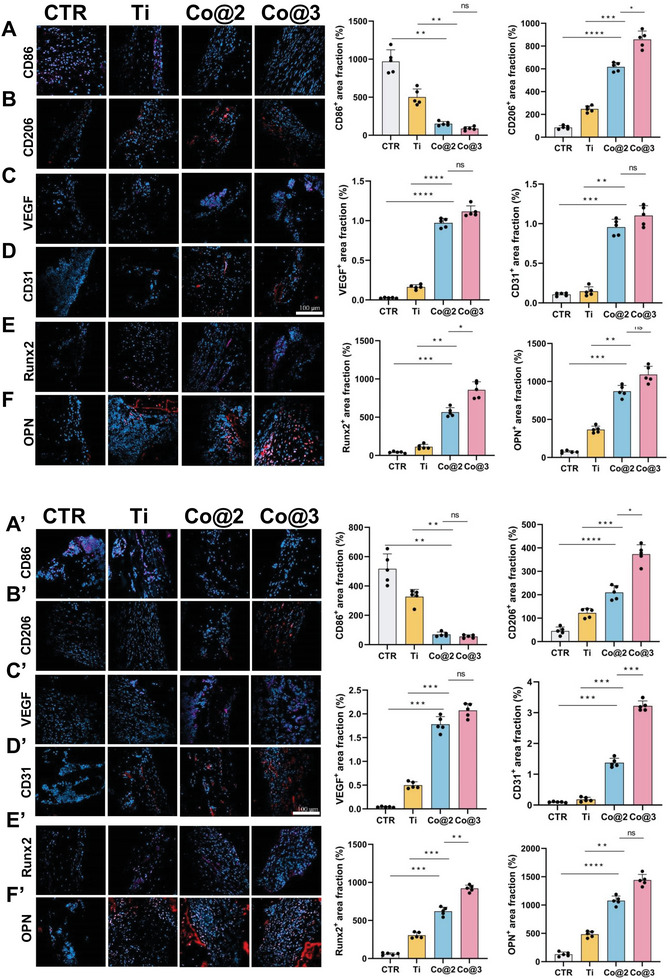
Immunofluorescence analysis of implantation sections post‐surgery. A–F) Immunofluorescence (IF) staining and quantitative analysis of specific markers at the 4th week post‐surgery for groups CTR, Ti, Co@2, and Co@3: A) CD86, B) CD206, C) VEGF, D) CD31, E) Runx2, F) OPN. A’–F’) Corresponding IF staining and quantitative analysis at the 8th‐week post‐surgery for the same markers. Scar bar = 100 µm. Error bars indicate standard deviation (SD), with *n* = 5 representing the number of samples per group. (*p < 0.05, **p < 0.01, ***p < 0.001, and ****p < 0.0001).

First, regarding immunomodulatory functions, by detecting the ratio changes of M1‐type (CD86‐positive) and M2‐type (CD206‐positive) macrophages, it was observed that cobalt‐containing implants (Co@2, Co@3) significantly reduced the number of M1‐type macrophages and increased the number of M2‐type macrophages (**Figure** **11**A,B,A’,B’). This moderated immune response is crucial for combating bacterial infections, promoting BMSC recruitment, and the osteogenic process. The immune response eventually decreased, and the groups treated with cobalt‐containing implants showed a noticeably quicker decline in immune response than those treated with pure titanium. This suggests that cobalt‐containing implants can successfully control the homeostasis of the microenvironment surrounding the implant by reducing pro‐inflammatory factors and increasing anti‐inflammatory factors.

Second, angiogenesis plays an essential role in the bone regeneration process. According to this study's immunofluorescence examination of CD31 and VEGF, the cobalt‐containing implant groups showed better angiogenic potential. CD31 and VEGF‐positive cells were increased in the Co@2 and Co@3 groups at 4 weeks post‐surgery, with more significant effects as the cobalt content increased (Figure 11C,D). By the 8th week, distinct circular mature vascular structures were observed in the Co@2 and Co@3 groups, indicating that implants incorporating cobalt have better angiogenic properties than scaffolds made of conventional titanium. The polarization of M2 macrophages and the release of Co^2+^ ions, which may encourage angiogenesis, are probably the causes of this enhanced vascularization (Figure 11C’,D’).^[^ ^31^, ^63^, ^64^ ^]^

Furthermore, to evaluate the osteointegration capacity of the implants, this study analyzed the expression of osteoblast‐related factors (OPN, Runx2). The results showed that in the Co@3 group, both the early 4th week and the later 8th week, the expression levels of OPN and Runx2 were significantly higher than in other groups, indicating a significant osteogenic promotion effect of cobalt‐containing implants (Figure 11E,F,E’,F’). Although the expression levels in the Co@2 group were slightly lower than in the Co@3 group, they also showed good osteogenic potential. These results are consistent with in vitro cell experiments and micro‐CT analysis, further confirming the superiority of cobalt‐containing implants in promoting osteointegration. The high expression levels of OPN and Runx2 in the Co@3 group in the 4th week indicate that cobalt‐containing implants began to promote osteogenesis early. This early osteogenic effect may be closely related to the immunomodulatory function of cobalt ion release, which regulates the inflammatory microenvironment, inhibits excessive inflammatory responses, and provides favorable conditions for the differentiation and mineralization of osteoblasts.^[^ ^31^, ^64^ ^]^ By the 8th week, the sustained high expression of OPN and Runx2 in the Co@3 group further confirmed the effectiveness of cobalt‐containing implants in promoting long‐term osteointegration. The bone tissue around the implant had formed a more mature and stable structure, indicating that cobalt‐containing implants promote early osteogenesis and support long‐term bone repair and reconstruction processes. In contrast, the pure Ti group showed relatively low expression levels of OPN and Runx2 at the 4th and 8th weeks, indicating limited osteogenic potential, which may not meet the strict requirements for osteointegration under infectious conditions, further highlighting the potential advantages of cobalt‐containing implants in the repair of IBDs repairs.

**Figure 12:**
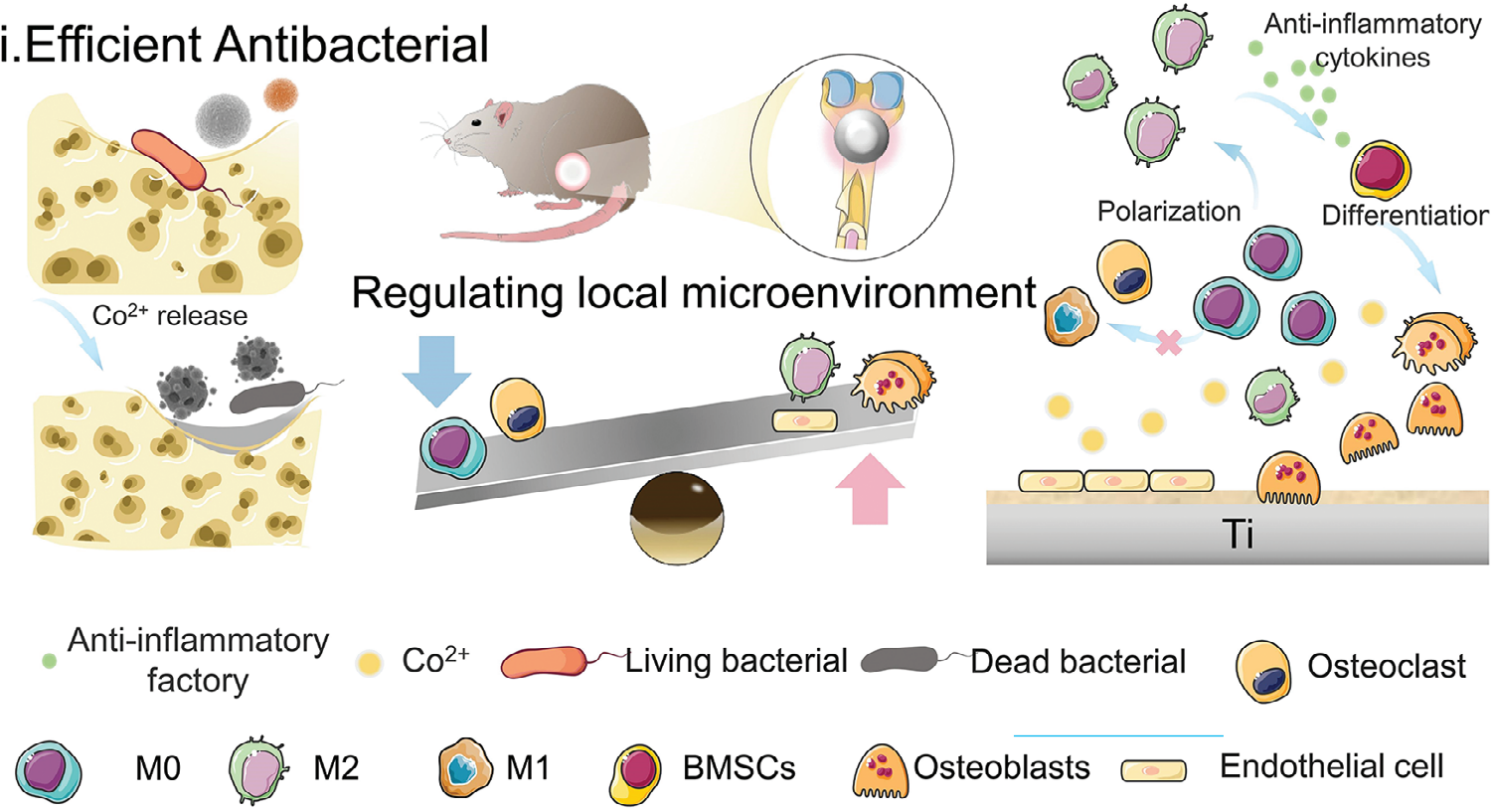
The diagrammatic representation delineates the mechanical action of cobalt‐bearing titanium (Co‐Ti) in facilitating the regeneration of infected bone defects within the biological system.

This study posits that cobalt‐containing implants, through the release of metal ions at the site of infectious bone defects, enhance the early bactericidal capacity, accelerate the resolution of immune responses, and promote the polarization of macrophages to the M2 type, thereby facilitating the occurrence of the osteogenic process (**Figure** **12**). This finding provides strong experimental evidence for the potential application of cobalt‐containing implants in the repair of infectious bone defects repairs and points the way for future clinical applications.

## 3 Conclusion

This work produced a unique orthopedic alloy material with outstanding bio‐physical adaptability to modulate immunological capacity and facilitate healing and tissue regeneration of infected bone deformities. This material has excellent physicochemical qualities, high cell compatibility, and strong antibacterial effects. It is created by integrating Co ions into a Ti alloy matrix. According to in vitro research, cobalt ion release promoted osteogenic differentiation synergistically and prevented osteoclast activation by triggering several signaling pathways related to bone resorption and formation. Furthermore, the altered substance caused macrophages to polarize toward an M2 phenotype that promotes healing, creating an immune milieu ideal for bone regeneration, and the material with cobalt ion implantation showed a lowering trend in TRAP enzyme activity. The cobalt‐containing substance successfully eliminates bacteria and other pathogens from rat bone infections. It also helps to polarize macrophages to the M2 phenotype, promoting osteogenic differentiation. Consequently, the cobalt‐containing titanium material presents a viable therapeutic option for healing infected bone lesions that is easy to use, less invasive, and does not require the induction of drug‐resistant antimicrobial agents or the exogenous addition of expensive biofactors.

In conclusion, it is anticipated that the cobalt‐containing titanium material described in this work will become a cutting‐edge therapeutic approach for managing bone infections. Additionally, this work clarifies how bone immunomodulation, a method of cobalt implantation, regulates the osteogenic differentiation process of BMSCs, offering a strong experimental platform for further investigations into bone repair materials. Moreover, the sequencing data obtained in this study reveal a potential mTOR‐Sema6d‐PPARγ pathway. Identifying this pathway adds a new molecular dimension to the proof of the scaffold's ability to modulate macrophage polarization, further substantiating our cobalt‐containing titanium material's therapeutic potential in bone repair and immune modulation.

## 4 Experimental Section

### Sample Preparation

Titanium discs measuring 15 × 15 × 1 mm were subjected to ultrasonic pretreatment to remove the oxide layer using a mixed acid solution (HF: HNO_3_: H_2_O = 1:5:34), followed by sequential ultrasonic cleaning with ethanol, acetone, deionized water, and ultrapure water. The samples were then air‐dried and designated as Ti. The Ti discs were placed in the PIII chamber. Co ions were implanted into the titanium metal surface according to the process parameters outlined in Table S1 (Supporting Information). The resulting samples were denoted as Co@1, Co@2, Co@3, and Co@4. For in vivo experiments, pure Ti cylinders with a diameter of 3 mm and a length of 5 mm were prepared using the same fabrication process as the titanium discs. All samples were sterilized with 75% alcohol for 2 h before biological evaluation.

### Surface Structure and Chemical Properties

Using a scanning electron microscope, the materials' surface morphology was investigated (SEM; S‐3400N, Hitachi, Japan). The surface's elemental composition was determined using energy‐dispersive spectroscopy (EDS, IXRF‐550i, IXRFSYSTEMS, USA). An X‐ray diffractometer (XRD; D8ADVANCE, Bruker, Germany) fitted with Cu‐Kα radiation (λ = 0.15406 nm) was used to determine the phase composition. AXISSupra, Kratos, UK, used X‐ray photoelectron spectroscopy (XPS) to evaluate the coatings' chemical structure. The surface roughness was examined using an atomic force microscope (AFM; MFP‐3D‐S; Asylum Research, USA).

### Assessment of Ion Release

PIII samples were incubated for 1, 4, 7, 14, 21, and 28 days at 37 °C without stirring in 5 mL of phosphate‐buffered saline (PBS). Following each immersion period, the ion release solution was gathered, kept at 4 °C, and replaced with brand‐new PBS. Inductively coupled plasma mass spectrometry (ICP‐MS; NuInstruments, UK) was used to measure the release of ions.

### Wettability Assessment

The water contact angles on the surfaces of various samples were measured with a contact angle instrument (SL200B, Solon, China). Ultrapure water (2 µL) was vertically dropped onto the sample surface, and the contact angle of each droplet was measured.

### Electrochemical Testing

Dynamic potential polarization curves of the samples were obtained in physiological saline solution (pH 7, 0.9 wt.% NaCl) using a CHI760C electrochemical workstation (Shanghai, China) at a scan rate of 10 mV s^−1^. A standard three‐electrode setup was used for the experiments, consisting of the sample acting as the working electrode, a graphite rod as the counter electrode, and a saturated calomel electrode as the reference electrode.

### Elastic Modulus Testing

The elastic modulus of the samples was measured using a Hysitron TS 77‐type nanoindentation tester (Bruker Corporation, Germany).

The data acquisition and control system had a displacement resolution of 0.01 nm, a time resolution of 0.005 s, a load resolution of 3 nN, and a maximum load force of 5000 mN. The loading, holding, and unloading times were set to 5, 2, and 5 s, respectively. The loading rate was 1 mN s^−1^. Five samples were measured per group, and the final results were presented as average values.

### Bacterial Inoculation

MRSA was injected onto the sample surfaces to assess the titanium disc surfaces' antibacterial qualities before and after ion implantation. Following sterilization using a 75% ethanol solution, the specimens were arranged in a 24‐well plate, with 60 µL of bacterial suspension at a 10^7^ CFU mL^−1^ density added to each well. After that, the plates were kept in a bacterial incubator set at 37 °C.

### Bacterial Plating

After incubating the samples, they were transferred into centrifuge tubes with 5 mm of physiological saline. The bacteria were then separated from the sample surfaces using vortexing, yielding a bacterial suspension. Following dilution, 100 µL of the MRSA solution was applied to standard Luria Bertani (LB) agar plates, carefully spaced with a glass rod, and allowed to incubate for 16 h at 37 °C in a bacterial incubator. A gel imaging system (Protein Simple, USA) was utilized to photograph the bacterial colonies, following which a count of the colonies was conducted. Based on this count, the antibacterial rate was then calculated.

### Anti‐Biofilm Activity Assay

MRSA cultured overnight in LB medium (1 × 10^6^ CFU mL^−1^) was inoculated into a 24‐well microtiter plate (500 µL per well) and incubated statically at 37 °C for 48 h to form biofilms. After further incubation with the samples for 2, 4, and 6 h, crystal violet (CV, 0.2% w/v) staining was used to quantify the bacteria within the biofilms.

### Bacterial Morphology

After 24 h of vaccination, 500 µL of a 2.5% glutaraldehyde solution was added to each well to fix the bacteria. The samples were then stored in a 4 °C refrigerator in the dark. Subsequently, the samples were dehydrated through a graded series of water/ethanol and ethanol/hexamethyldisilazane solutions, air‐dried, and observed for bacterial morphology under a scanning electron microscope.

### In Vitro Cell Culture

Mouse monocyte‐macrophage leukemia cells (RAW264.7; Shanghai Cell Bank, Chinese Academy of Sciences) were grown in DMEM media supplemented with 10% fetal bovine serum (FBS, Gibco, USA) and 1% penicillin‐streptomycin (HyClone, USA). In Minimum Essential Medium Alpha (α‐MEM, Corning, USA) supplemented with 10% FBS and 1% penicillin‐streptomycin, mouse bone marrow mesenchymal stem cells (BMSCs; Shanghai Cell Bank, Chinese Academy of Sciences) were cultured. The two types of cells were grown in a 37 °C, 5% CO_2_ humidified incubator. The growing media (osteogenic medium, OM) was added with β‐glycerophosphate (10 mm), L‐ascorbic acid (50 g mL^−1^), and dexamethasone (10 nm) to investigate the osteogenic differentiation of BMSCs. 1 µg mL^−1^ of LPS was introduced into the culture medium to induce an inflammatory milieu and activate macrophages.

### Cell Biocompatibility

The biocompatibility of the samples was tested using a Live/Dead staining kit (Beyotime, China) and a Cell Counting Kit‐8 (CCK‐8; Beyotime, China).

### In Vitro Immunological Assessment

RAW264.7 cells were used to test the substances' anti‐inflammatory properties. RAW264.7 cells were planted at a density of 1 × 10^4^ cells per well in a 24‐well plate to examine the polarization of macrophages. Following an overnight culture, 1 µg mL^−1^ LPS was added to the culture media to activate the macrophages for 3 h. After that, the cells underwent three PBS rinses before being cultured in conditioned media. Cells treated with LPS or left untreated were positive and negative controls, respectively.

Using immunofluorescence labeling, macrophage polarization was observed after both 4‐ and 7‐day cultures. The cell nuclei were counterstained with DAPI after the cells were stained with antibodies against CD86 and CD206. A 3DHISTECH Panoramic MIDI was used to record fluorescence images. Griess assay kit (Beyotime Biotechnology S0021) was used to quantify NO release during the cell culture phase while culture supernatants were collected from each well concurrently.

RNA quality was assessed using an Agilent 2100 Bioanalyzer (Agilent Technologies, Palo Alto, CA, USA) for RNA sequencing. Majorbio Co., Ltd. sequenced qualified samples using the Illumina platform, utilizing three biological replicates for each group. Bioinformatics techniques were used to examine the RNA‐seq data, with a threshold set for fold changes ≥2 (upregulated or downregulated). Total RNA was extracted from each sample, and gene expression of mTOR, Sema6d, PPARγ, CD206, and CD86 was analyzed by quantitative reverse transcription polymerase chain reaction (RT‐qPCR, QuantStudioTM3, ThermoFisher). Data were analyzed using the 2−ΔΔCt method and normalized to the negative control group to determine the relative gene expression levels. Primer sequences are listed in Table S2 (Supporting Information).

### Osteogenic Differentiation Assessment

To evaluate the osteogenic differentiation capacity, alkaline phosphatase (ALP) activity was measured using an ALP assay kit (Beyotime, China) at 7‐ and 14‐days post‐culture. The transformation of p‐nitrophenyl phosphate to p‐nitrophenol, catalyzed by ALP activity, was determined by absorbance at 405 nm using a microplate reader. The ALP levels were adjusted to the total protein content using a bicinchoninic acid (BCA) protein assay kit.

For histologically staining ALP activity, stem cells on the sample surfaces were stained using a BCIP/NBT kit (Beyotime‐Biotech Co., China). Using the BCIP/NBT working solution, the staining process was carried out for 30 min at room temperature in the dark.

Alizarin Red S staining evaluated the calcium mineralization of BMSCs co‐cultured with the materials. Following a 21‐day culture period, the cells underwent three PBS washes, were fixed for 15 min in 95% (v/v) ethanol, and then underwent one more PBS wash using distilled water. The entire surface of the scaffold was then coated with 0.1% (w/v) Alizarin Red S solution that had been adjusted to pH 4.2. Ultimately, cetylpyridinium chloride in 10% (w/v) 10 mm sodium phosphate (pH 7.0) was used to quantify the mineralized nodules, and a microplate reader was used to detect the absorbance at 562 nm.

Reverse transcription polymerase chain reaction (RT‐PCR) was used to assess the expression of osteogenic genes, such as COL1, OPN, and Runx2, on day 7 of incubation. The outcomes were adjusted to match the control group, which consisted of blank culture plate‐cultured cells. Table S2 (Supporting Information) contains a list of primer sequences.

### Osteoclast Detection

Samples were co‐cultured with RAW264.7 cells for 4 and 7 days, after which tartrate‐resistant acid phosphatase (TRAP) activity was measured using a TRAP Assay Kit (Beyotime, China).

On the 4th day of incubation, the expression of osteoclastogenic genes such as TRAP, DC‐STAMP, CTSK, and c‐Fos was assessed using reverse transcription polymerase chain reaction (RT‐PCR). The data were standardized relative to the control group, which consisted of cells cultivated on a blank culture plate. The primer sequences utilized in this investigation are described in Table S2 (Supporting Information).

### Rat Infected Femoral Defect Model

The Xiangya School of Medicine, Central South University, granted ethical approval for all in vivo investigations under approval number XMSB‐2022‐000, guaranteeing adherence to the ARRIVE criteria at every stage. The rats were fed separately and kept in rooms at room temperature. To prevent postoperative infection, all rats received injections of penicillin after surgery and were kept under general anesthesia the entire time.

Four groups of five male Sprague Dawley rats (250–300 g) were randomly assigned: control, Ti, Co@2, and Co@3. A critical‐sized defect (diameter 3 mm, depth 5 mm) was drilled under isoflurane gas anesthesia and lidocaine local anesthetic in the femur's diaphysis. To mimic an infected bone defect, *Staphylococcus aureus* cells in PBS (20 µL, 1 × 10^7^ CFU mL^−1^) were injected into the defect. Afterward, sterile forceps were used to implant Ti, Co@2, and Co@3 samples into the rat bone defects. The skin and joint capsule were sutured layer by layer following ligament removal. Rats were euthanized on weeks 4 and 8 post‐implantation, and samples were collected from their knees.

### Blood Co^2+^ Analysis

Blood samples were collected from the rats 2 months after implantation, with at least 1 mL of blood taken from each rat. Inductively coupled plasma mass spectrometry (ICP‐MS) was used to determine the blood samples' metal ions (cobalt) content. The trend of metal ion content in the blood was analyzed according to standard methods to assess the durability and biocompatibility of metal hip implants and their potential impact on the rats.

### In Vivo Bio‐Safety

After 8 weeks, the rats with implanted materials were euthanized. Organ tissues (heart, liver, spleen, lungs, and kidneys) were collected, fixed, embedded, and stained with hematoxylin and eosin (H&E) to observe pathological changes.

### In Vivo Antibacterial Activity and Bone Regeneration

Tibial sections were stained with Giemsa solution to evaluate the antibacterial capacity of the materials, and the staining results were observed under a light microscope (IX73, Olympus). All images of new bone regeneration around the defect area were obtained using a micro‐CT system (NEMO NMC‐200, PINGSENG SCIENTIFIC), and the bone repair capacity was qualitatively assessed using Masson's staining and HE staining.

### Immunofluorescence Staining

To further evaluate the expression of osteogenic markers (Runx2, OPN), angiogenic markers (VEGF, CD31), and polarization markers (CD86, CD206), immunofluorescence staining was performed.

### Statistical Analysis

Data were presented as mean ± standard deviation. All experiments were repeated three times. Multiple comparisons were assessed using one‐ or two‐way analysis of variance (ANOVA) with GraphPad Prism 7.0. The level of statistical significance was set at *p* < 0.05, where ns indicates insignificant. *p < 0.05, **p < 0.01, ***p < 0.001.

## Conflict of Interest

The authors declare no conflict of interest.

## Supporting information



Supporting Information

## Data Availability

Research data are not shared.
