# Correction to “A Multifunctional Cobalt‐Containing Implant for Treating Biofilm Infections and Promoting Osteointegration in Infected Bone Defects Through Macrophage‐Mediated Immunomodulation”

**DOI:** 10.1002/advs.202502472

**Published:** 2025-02-22

**Authors:** Nongyang Yan, Hao Zhou, Jinpeng He, Tengfei Li, Qi Liu, Hao Ning, Zhixin Ma, Linfei Feng, Tao Jin, Youwen Deng, Zhengwei Wu, Shauna Celeste Kennard

https://doi.org/10.1002/advs.202409200

In the original publication, the image representing the Co@3 group was mistakenly shown in Figure 4C with the correct image as follows:


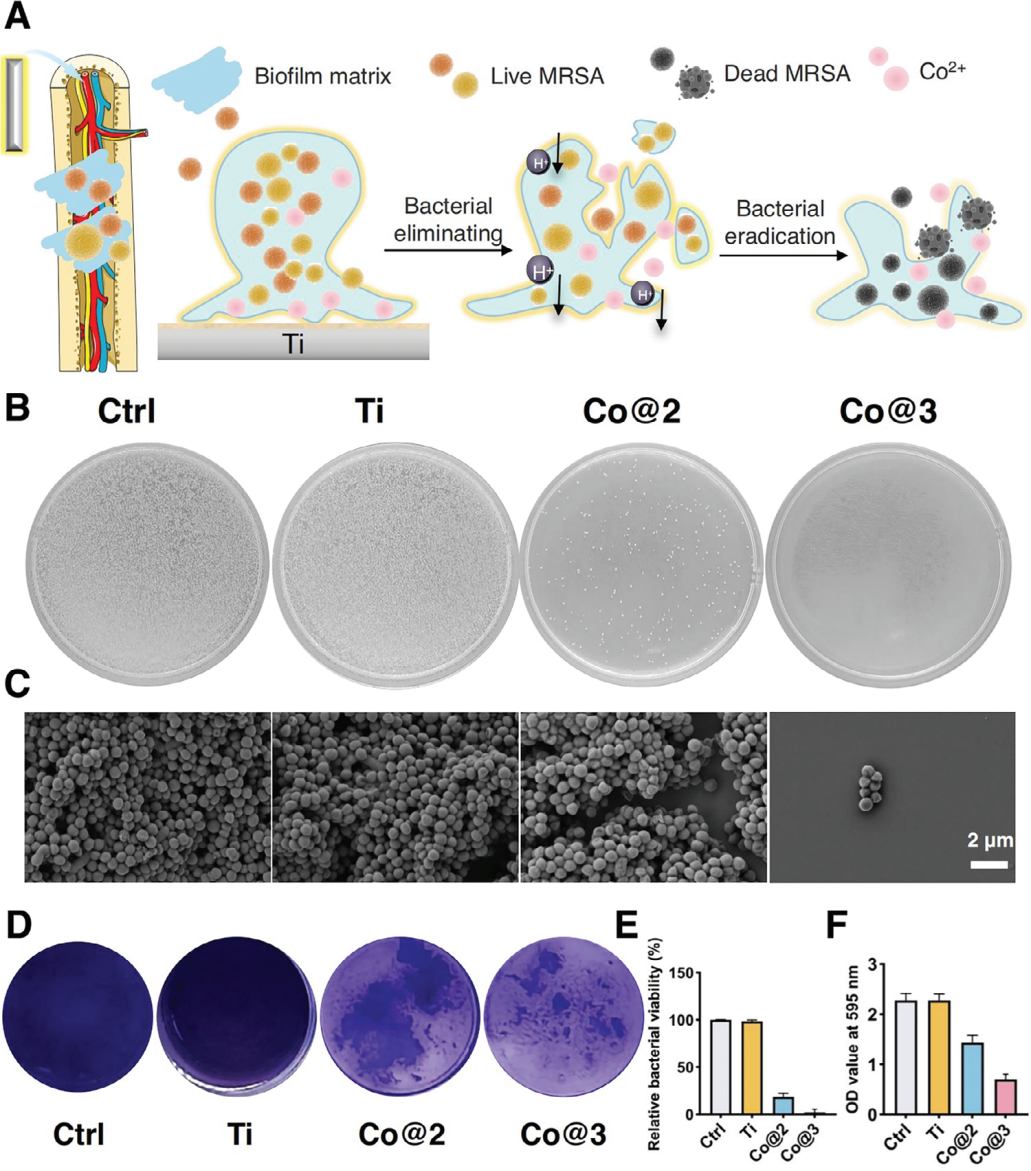


Figure 4 Co‐Ti effectively eliminates MRSA biofilm. A) Schematic diagram of the antibiofilm mechanism of Co‐Ti, illustrating the transition from biofilm matrix disruption to bacterial elimination and eradication. B) MRSA colonies visualized on agar plates after treatment with different samples: Control, Ti, Co@2, and Co@3.C) SEM images of MRSA biofilms following incubation with different samples, demonstrating morphological changes and bacterial removal. Scale bar = 2 µm. D) Crystal violet staining of biofilm biomass after treatment with Control, Ti, Co@2, and Co@3, showing reduced biofilm formation in Co@3‐treated samples. E) Relative bacterial viability of MRSA after treatment with various samples, quantified as a percentage. F) Quantification of biofilm biomass at OD 595 nm after treatment with different samples, with results shown as mean ± SD (*n* = 3).

We apologize for this error.

