# Supplementary material for: A Multifunctional Cobalt‐Containing Implant for Treating Biofilm Infections and Promoting Osteointegration in Infected Bone Defects Through Macrophage‐Mediated Immunomodulation
Source: Adv Sci (Weinh). 2024 Nov 26;12(3):2409200. doi: 10.1002/advs.202409200 (PMC11744729; doi:10.1002/advs.202409200)
Supplement: Supplementary file 1 — Supporting Information [file ADVS-12-2409200-s001.docx]

1. **Supplementary Materials and Methods**

1.1 Cell Proliferation and Viability

Cells were cultivated for 1, 4, and 7 days after being seeded on the samples at a density of 1×10^5^ cells per well (in quadruplicate). Cell growth and viability were measured using a Cell Counting Kit (CCK-8) (Beyotime, Shanghai, China) in a microplate reader (Tecan, M200pro, Switzerland) at 450 nm after each incubation period. After staining the cells for 30 minutes at 37°C using a Live/Dead staining working solution (Dojindo Molecular Technologies, Japan), the cells were examined with a laser confocal microscope (Leica TCSSP8, Germany).

1. **Supplementary Figures and tables.**

**Table S1.** PIII parameters for metals.

| Type of metal | Decel power supply(KV) | Decel power supply(KV) | Target voltage (kV) | Target frequency (Hz) | Target pulse width (μs) |
| --- | --- | --- | --- | --- | --- |
| Co | 1.5 | 30 | 20 | 3 | 2000 |

**Table S2.** Primer sequences for qRT-PCR analysis.

| Runx-2 Forward Sequence | TCGTCAGCGTCCTATCAGTTCC | Rat |
| --- | --- | --- |
| Runx-2 Reverse Sequence | CTTCCATCAGCGTCAACACCATC | Rat |
| COL-1α Forward Sequence | GTGCGATGGCGTGCTATGC | Rat |
| COL-1α Reverse Sequence | CTATGACTTCTGCGTCTGGTGATAC | Rat |
| OPN Forward Sequence | AGCAAGAAACTCTTCCAAGCAA | Rat |
| OPN Reverse Sequence | GTGAGATTCGTCAGATTCATCCG | Rat |
| TRAP Forward Sequence | CACTCCCACCCTGAGATTTGT | Rat |
| TRAP Reverse Sequence | CATCGTCTGCACGGTTCTG | Rat |
| DC-STAMP Forward Sequence | AAAACCCTTGGGCTGTTCTT | Rat |
| DC-STAMP Reverse Sequence | AATCATGGACGACTCCTTGG | Rat |
| CTSK Forward Sequence | GGATGAAATCTCTCGGCGTTT | Rat |
| CTSK Reverse Sequence | GGTTATGGGCAGAGATTTGCTT | Rat |
| c-Fos Forward Sequence | CCAAGCGGAGACAGATCAACTT | Rat |
| c-Fos Reverse Sequence | TCCAGTTTTTCCTTCTCTTTCAGCAGAT | Rat |
| PPARγ Forward Sequence | TCGCCAAGGTGCTCCAGAAG | Rat |
| PPARγ Reverse Sequence | AGGCTCATATCTGTCTCCGTCTTC | Rat |
| sema6d Forward Sequence | GCCAGGGAGTGTGTGAGAGAG | Rat |
| sema6d Reverse Sequence | CGTGTTGCCATACTCGGTGTC | Rat |
| mTOR Forward Sequence | TGGCAAGAGCGGCAGACTG | Rat |
| mTOR Reverse Sequence | GTTGGCAGAGGATGGTCAAGTTG | Rat |
| CD86 Forward Sequence | AACGTATTGGAAGGAGATTACAGCT | Rat |
| CD86 Reverse Sequence | CCTGCTAGGCTGATTCGGCT | Rat |
| CD206 Forward Sequence | TTGGACGGATAGATGGAGGGT | Rat |
| CD206 Reverse Sequence | CCATAGAAAGGAATCCACGCA | Rat |


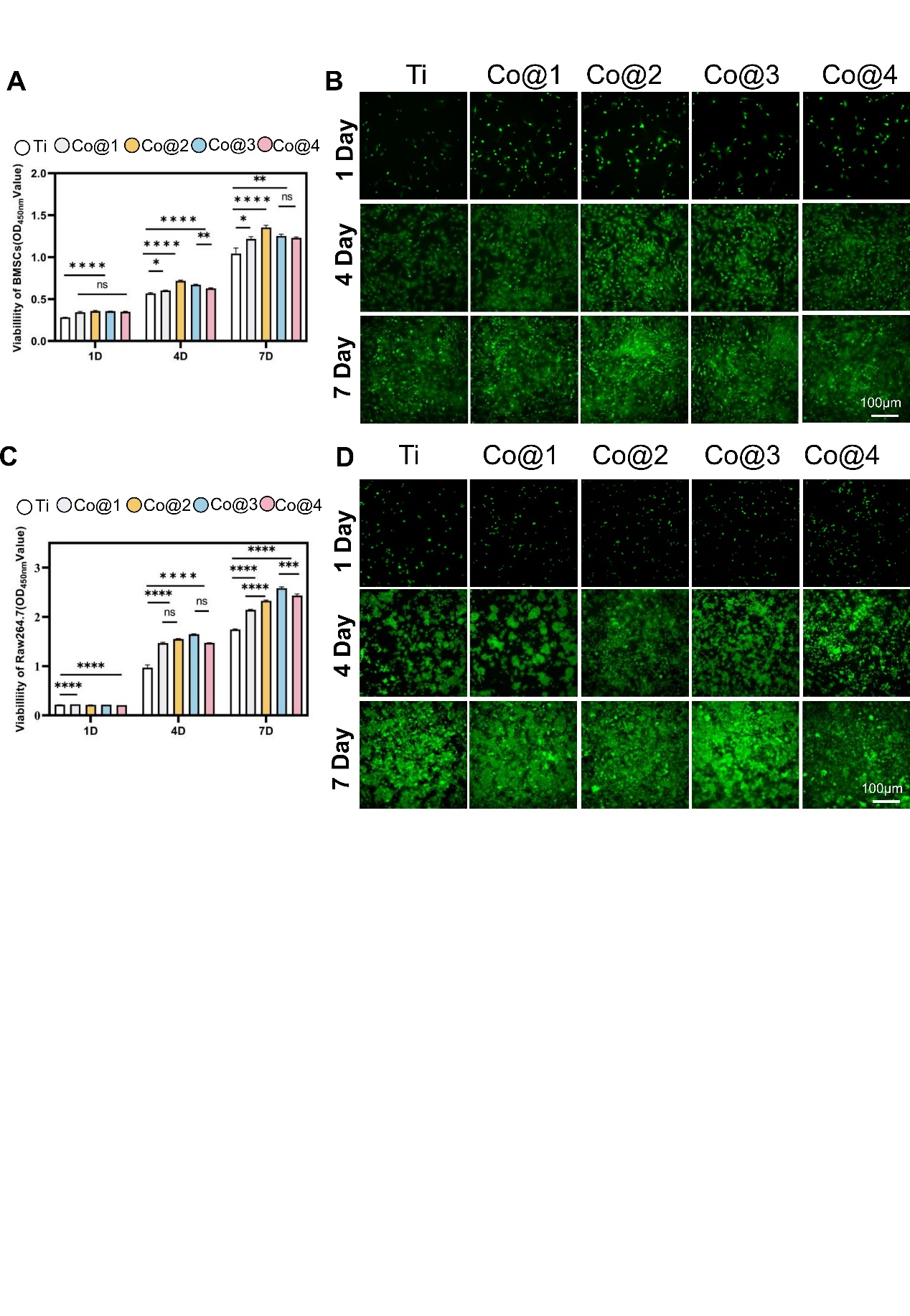


Fig S1 Sample Surface Morphology and Cell Viability. A) CCK-8 assay measuring the vitality of BMSCs over 1, 4, and 7 days on various sample surfaces. B) Staining BMSC morphology live/dead using different samples for 1, 4, and 7 days. C) RAW264.7 vitality measured by the CCK-8 test across 1, 4, and 7 days on various sample surfaces. D) Staining RAW264.7 morphology live/dead with different samples for 1, 4, and 7 days (n=4). (ns means not significant, **p < 0.01, ***p < 0.001, and ****p < 0.0001.)


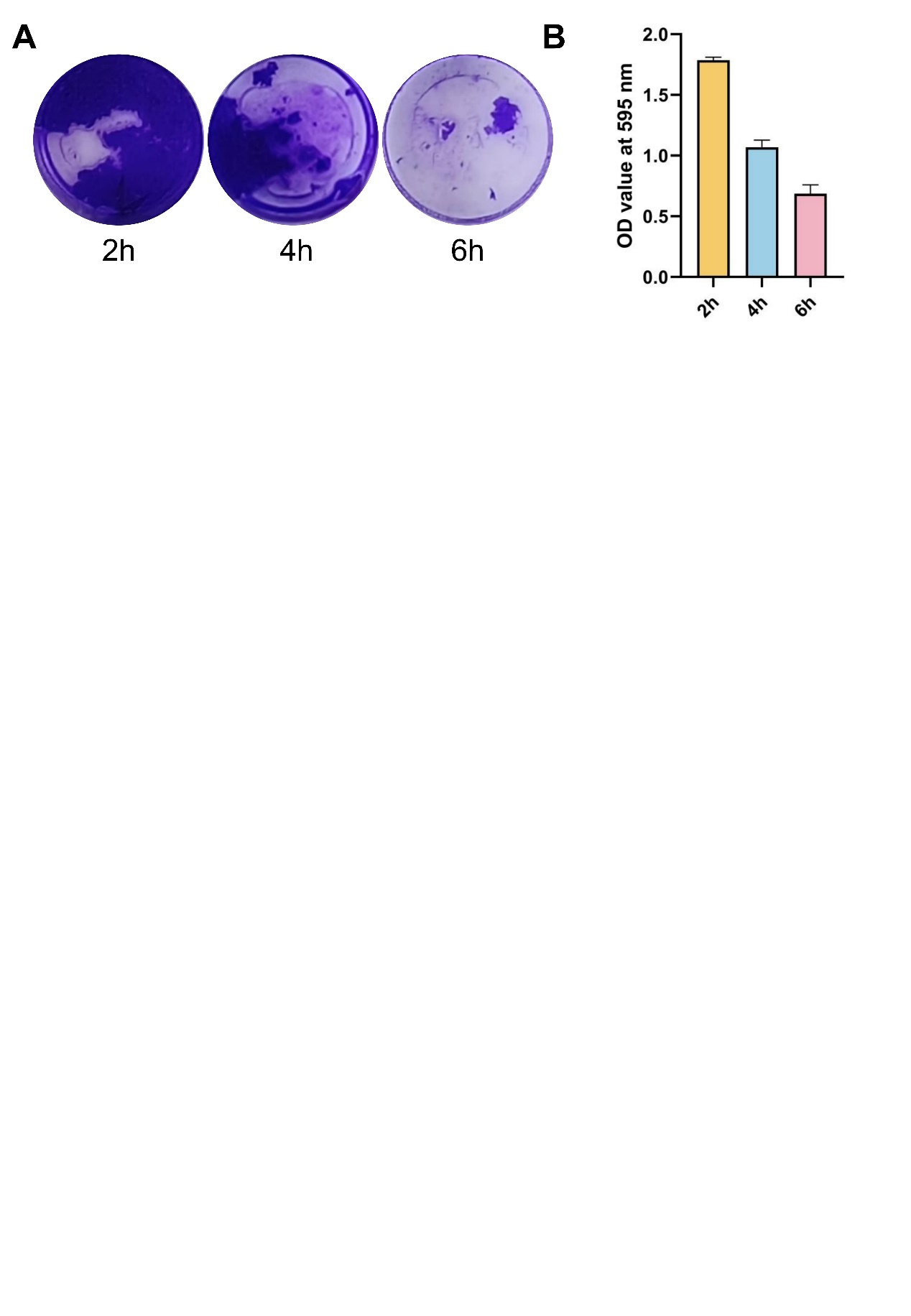


Fig S2. . Time-dependent inhibition of MRSA biofilm by Co@3 treatment. A) Crystal violet staining of MRSA biofilm treated with Co@3 at different time points (2 h, 4 h, 6 h). B) Quantification of biofilm biomass, represented by OD value at 595 nm, indicating a gradual decrease in biofilm formation with increasing treatment time. Data are presented as mean ± standard deviation (SD) (n = 3).


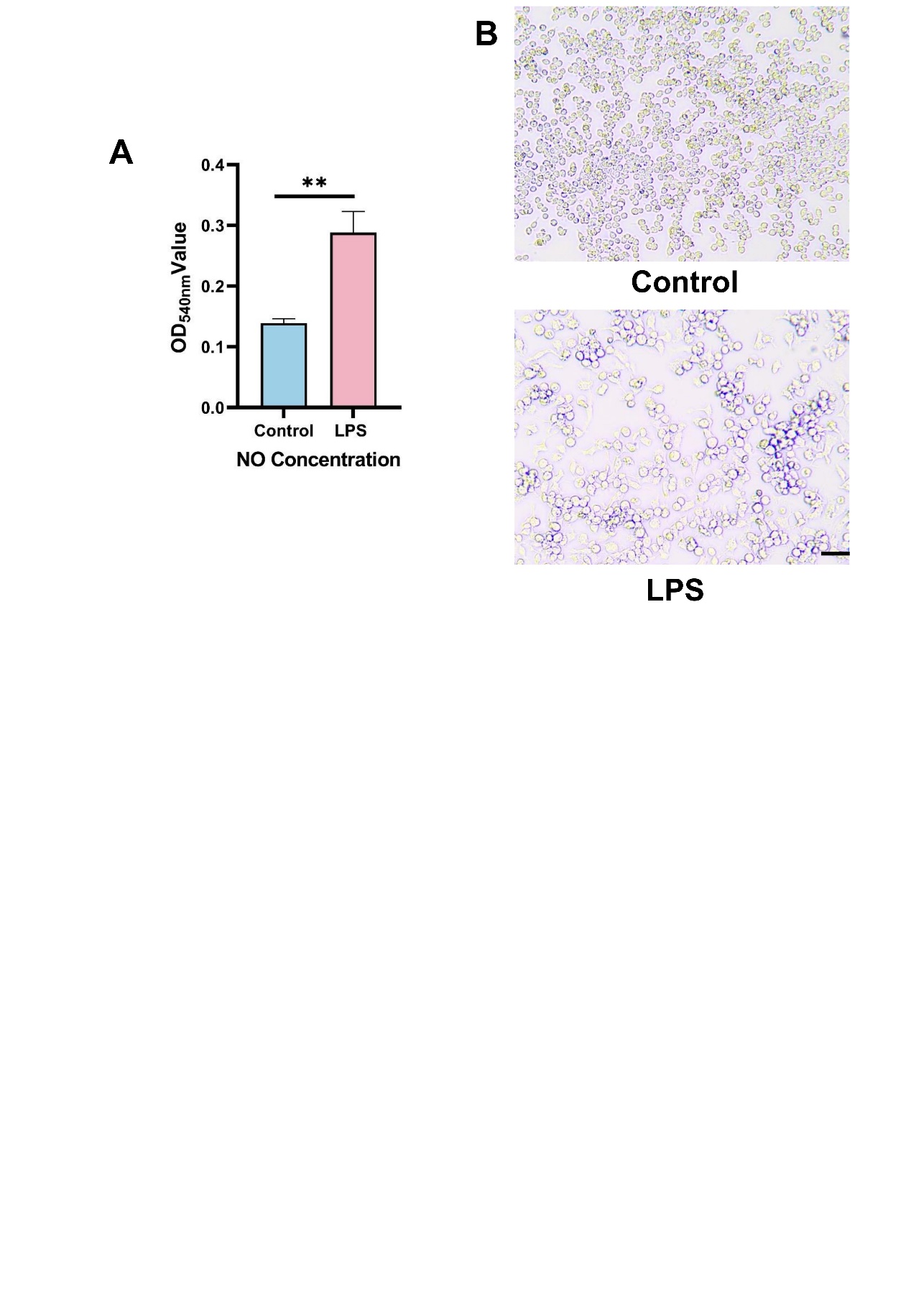


Fig S3. A) The NO content was determined by Griess reagent. B) Photographs of RAW264.7 cells were treated with LPS 3 h under optical microscopy (n=4). Scale bar = 50μm. (**p < 0.01.)


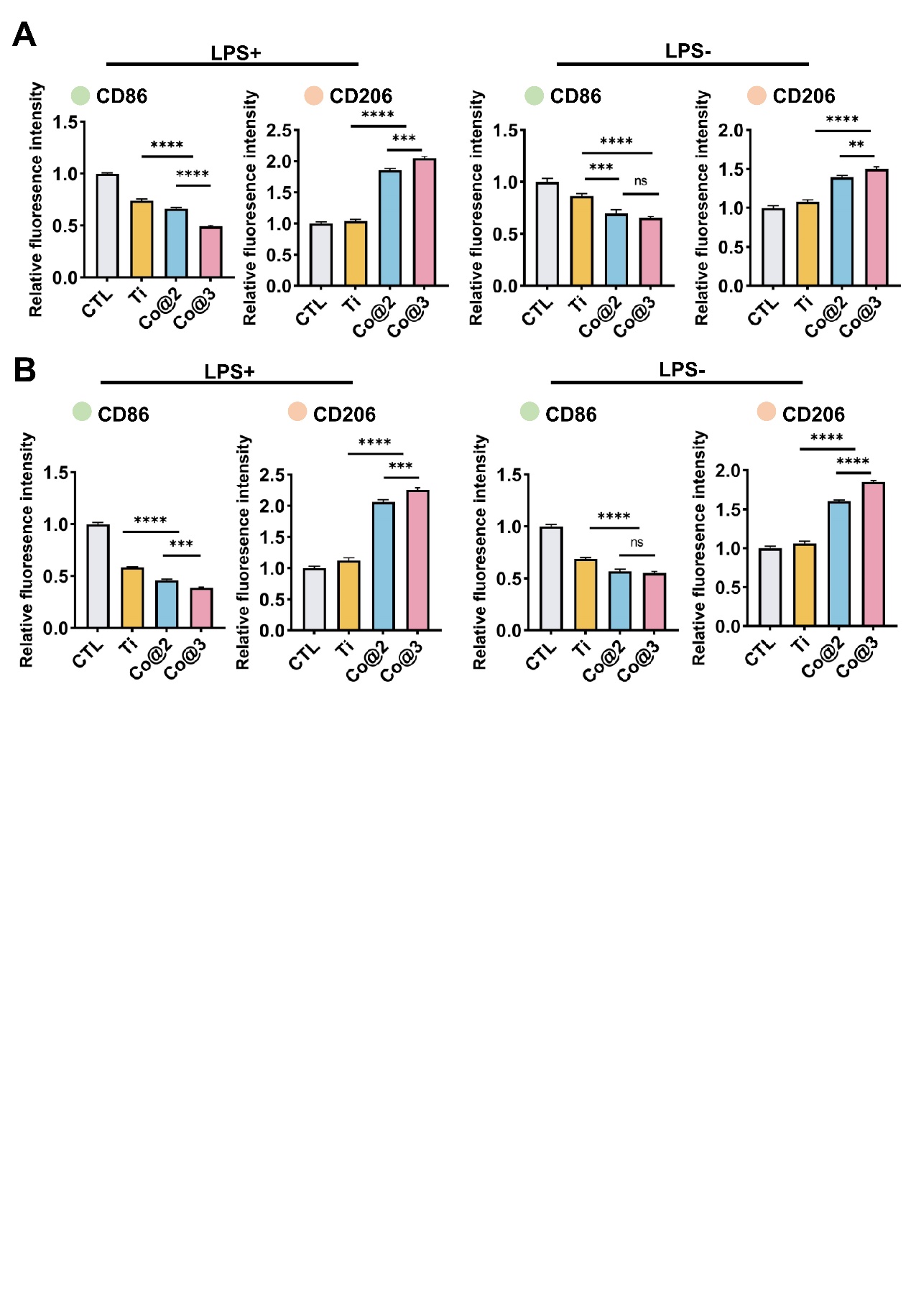


Fig S4. A) Quantitative analysis of fluorescence microscopy images showing the expression levels of CD86 and CD206 in RAW264.7 cells cultured with the samples on day 4. B) Quantitative analysis of fluorescence microscopy images showing the expression levels of CD86 and CD206 in RAW264.7 cells cultured with the samples on day 7 (n=3). (ns means not significant, **p < 0.01, ***p < 0.001, and ****p < 0.0001.)


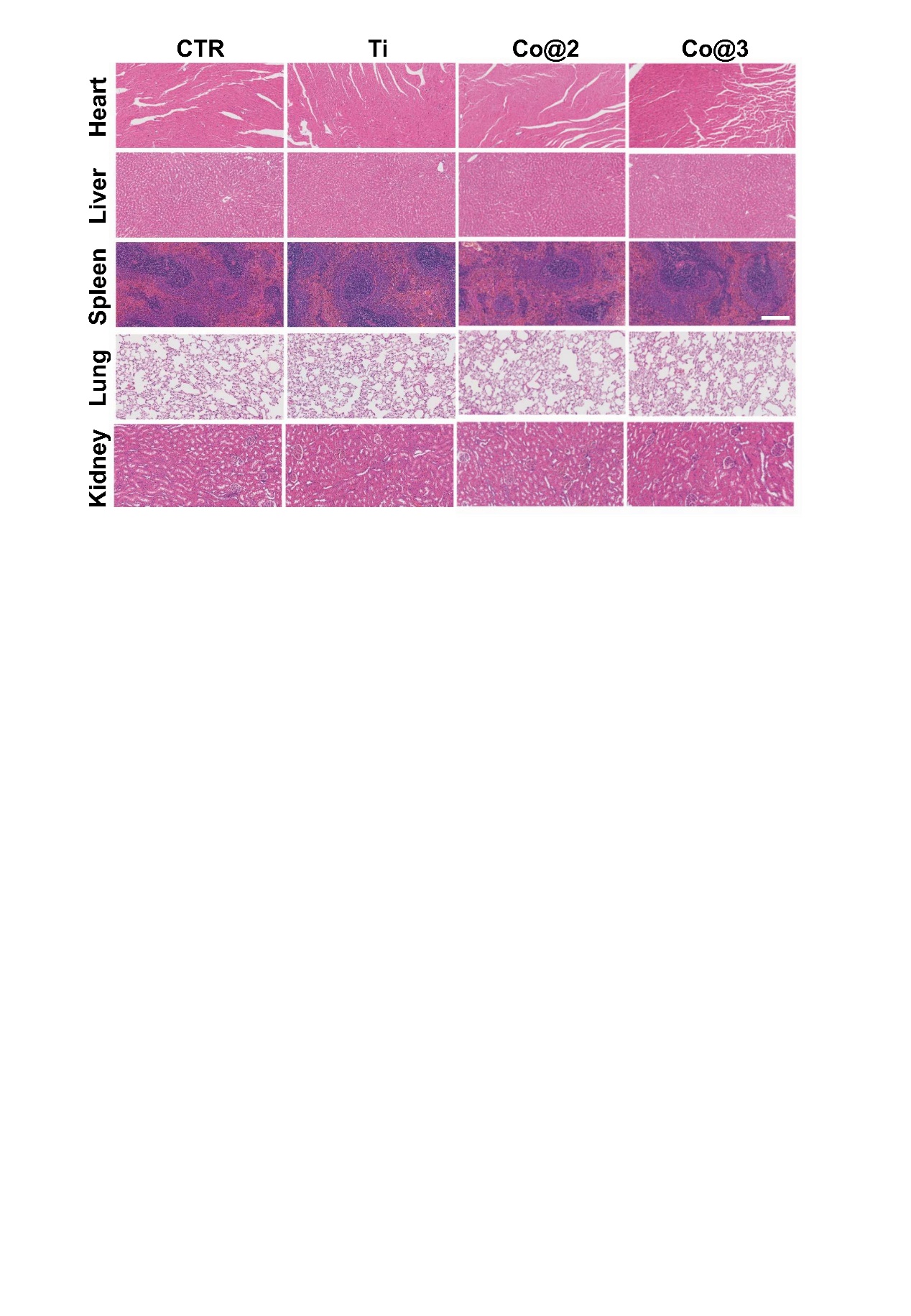


Fig S5. H&E staining of vital organ tissues from rats at 8 weeks post-surgery, including the heart, liver, spleen, lung, and kidney, to evaluate histological characteristics. Scale bar = 200μm.


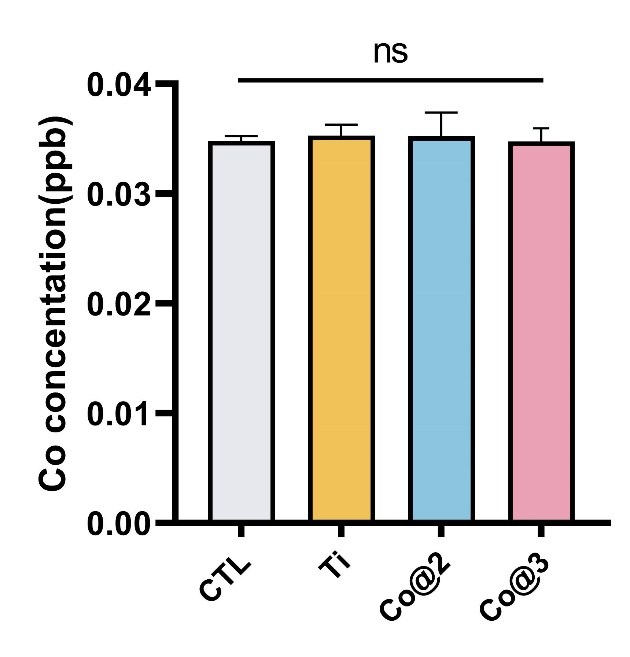


Fig S6. Cobalt Ion Concentration in Rat Blood Measured by ICP (n=3). (ns means not significant.)
